# A multidimensional classification of public health activity in Australia

**DOI:** 10.1186/1743-8462-6-9

**Published:** 2009-04-09

**Authors:** Louisa Jorm, Su Gruszin, Tim Churches

**Affiliations:** 1 School of Medicine, University of Western Sydney, Campbelltown Campus, Locked Bag 1797, Penrith South DC, NSW 1797, Australia; 2 Public Health Information Development Unit, The University of Adelaide, Level 9, 10 Pulteney St, Adelaide, SA 5005, Australia; 3 Centre for Epidemiology and Research, New South Wales Department of Health, Locked Mail Bag 961, North Sydney, NSW 2059, Australia

## Abstract

**Background:**

At present, we have very limited ability to compare public health activity across jurisdictions and countries, or even to ascertain differences in what is considered to be a public health activity. Existing standardised health classifications do not capture important dimensions of public health, which include its functions, the methods and interventions used to achieve these, the health issues and determinants of health that public health activities address, the resources and infrastructure they use, and the settings in which they occur. A classification that describes these dimensions will promote consistency in collecting and reporting information about public health programs, expenditure, workforce and performance. This paper describes the development of an initial version of such a classification.

**Methods:**

We used open-source Protégé software and published procedures to construct an ontology of public health, which forms the basis of the classification. We reviewed existing definitions of public health, descriptions of public health functions and classifications to develop the scope, domain, and multidimensional class structure of the ontology. These were then refined through a series of consultations with public health experts from across Australia, culminating in an initial classification framework.

**Results:**

The public health classification consists of six top-level classes: public health 'Functions'; 'Health Issues'; 'Determinants of Health'; 'Settings'; 'Methods' of intervention; and 'Resources and Infrastructure'. Existing classifications (such as the international classifications of diseases, disability and functioning and external causes of injuries) can be used to further classify large parts of the classes 'Health Issues', 'Settings' and 'Resources and Infrastructure', while new subclass structures are proposed for the classes of public health 'Functions', 'Determinants of Health' and 'Interventions'.

**Conclusion:**

The public health classification captures the important dimensions of public health activity. It will facilitate the organisation of information so that it can be used to address questions relating to any of these dimensions, either singly or in combination. The authors encourage readers to use the classification, and to suggest improvements.

## Background

One of the principal ways in which we make sense of the world is to group things and events into classes that share common characteristics. Human beings learn to do this intuitively in early childhood, and quickly develop an understanding of classes for commonly encountered objects and concepts which is shared by all those around them. However, for more specialised areas, the description of things or events in terms of classes tends to be a far less intuitive process that demands a carefully thought-out, explicitly articulated framework. Such classification frameworks make it much easier to compare information about entities and concepts, and to discern their similarities and differences.

Within the domain of public health, little conceptual work has been done to develop shared definitions, terminologies or classifications. As a result, we have limited ability to compare public health activity across jurisdictions and countries, or even to ascertain whether we share common notions of what constitutes 'public health'. This in turn hinders our ability to collect comparable, time-series data on expenditure, workforce, or performance, and to set and monitor benchmarks for these.

In Australia, the governments of the six states and two territories are the major providers of public health services, while the responsibility for funding these services is shared between the Australian (national) Government and state and territory governments [1]. Local government (municipal and shire councils) also plays a role in delivering public health services, particularly in the areas of environmental health, urban planning, food safety and immunisation; this role varies among the states and territories.

The National Public Health Partnership, a body set up in 1996 to strengthen collaboration between the Australian Government and state and territory governments, adopted the following definition for public health:

*the organised response by society to protect and promote health, and to prevent illness, injury and disability. The starting point for identifying public health issues, problems and priorities, and for designing and implementing interventions, is the population as a whole, or population sub-groups*[2].

However, in Australia – as in other countries [3] – the term 'public health' is a source of confusion, because it is also often used to refer to health services provided by the state or otherwise paid for by taxpayers out of "the public purse", as opposed to services provided by the private sector, or paid for by individuals or nongovernmental health insurance or health maintenance funds. Some jurisdictions use the alternative term 'population health' to refer to the same domain, but this term is also poorly understood.

The lack of widespread understanding about what constitutes public health hampers efforts to advocate for more resources for the sector. If we as public health practitioners cannot clearly describe the activities of our sector, the resources expended by it, and its outcomes, it will remain difficult to convince the public, politicians and other decision-makers that greater investment is needed [4].

The lack of basic conceptual development within public health has been recognised internationally. An expert panel convened by the United States (US) Centres for Disease Control in 1999 identified the use of common definitions and comparable data sources as being among the most important issues for achieving the goal of quality improvement in public health through performance measurement [5]. Five years later, lack of terminological and conceptual consensus was cited as obstructing even basic work in the area of public health finance in that country [6]. In Australia, a 2002 project that set out to develop a key set of performance indicators for public health practice recommended, as a priority, the development of a common classification system that could be used for measuring expenditure as well as for organising performance measurement activities [7]. A 2003 review of the financing of population health (defined as a subset of public health with a whole-of-population focus) in eight Organisation for Economic Co-operation and Development (OECD) countries, noted that comparability was hampered by differing definitions and categorisations of activity, lack of reliable data, and lack of uniformity in methods for extracting information [8].

Several conceptual models describing 'core' or 'essential' functions of public health exist, including the framework described in the Institute of Medicine's 1988 report on the status of public health in the US [9], the '10 essential public health services' proposed by an expert panel convened by the US Department of Health and Human Services [10], the Australian National Public Health Partnership's 'core functions for public health practice' [2], a set of core functions promulgated by the Chief Medical Officer in the UK [11], and another developed by the World Health Organization (WHO) in 1996 [12] as well as 'essential public health functions' developed from a three-country study in WHO's Western Pacific Region in 2003 [13]. The Pan American Health Organization developed 'essential public health functions' and public health 'roles' in a conceptual renewal of public health in 2002 [14], and revised these in 2007 [15]. A list of 'essential functions' was recommended by the Canadian National Advisory Committee on Population Health in 2003 [16] (See Additional file 1). These conceptual models, in particular the '10 essential public health services', have proved valuable for deriving performance indicators, standards and associated measurement instruments [17-23]. However, all are essentially 'flat' lists, or at best hierarchical taxonomies, which conflate discrete dimensions such as the purpose of public health activities, the health issues and problems addressed and the settings in which services are delivered, into single 'functions'. None presents a well-defined theoretical framework for multiple aspects of effective public health practice [24].

Some of the many standard classifications that are already in use in health fields address aspects of public health. For example, WHO's international classifications of diseases [25], functioning and disability [26], and external causes of injury [27] can be used to classify morbidity and mortality data in terms of diseases, disability and injury of interest to public health. The recently created OECD system of health accounts [28,29] classifies health care in three dimensions for the purposes of international comparisons of health care spending: sources of funding; service providers; and functions of care (the goals or purposes of health care; e.g. disease prevention, health promotion). While these dimensions are clearly separate, the functional activity category of 'Prevention and public health services' [29] consists of a list of only six, non-exclusive, items (see Additional file 1).

A multidimensional approach was adopted by the Eastern Region Public Health Observatory in the United Kingdom (UK), for the construction of their Public Health Information Tagging Standard (PHITS). PHITS was developed to categorise and provide structure to information provided on websites, and to improve the efficiency of the retrieval of web-based public health resources. PHITS has seven dimensions: 'Person'; 'Time'; 'Place'; 'Determinants'; 'Morbidity and Mortality'; 'Services'; and 'Policy' [30]. It has now been integrated into a UK National Public Health Language thesaurus [31].

PHITS was designed primarily to categorise web-based information resources, rather than as a multi-purpose classification for public health. Like other existing classifications, it does not capture all the important dimensions of public health, which include its functions, the methods and interventions used to achieve these, the health issues and determinants of health which public health activities address, the resources and infrastructure they use, and the settings in which the activities occur.

A multidimensional classification of public health that describes all these dimensions and their relationships, and adopts elements from existing classifications where appropriate, will serve multiple purposes. It will have utility for standardising the collection of information about public health programs, expenditure, workforce and performance. It will facilitate aggregate reporting and analysis of this information in ways that suit particular perspectives; for example, according to the health problem addressed, or the setting where public health activity occurred. This paper presents an initial version of such a multidimensional classification, and describes the process that we used to develop it [32].

## Methods

We used an ontology-building process to develop the public health classification. The term "ontology" is used in several ways, but here we use it in the computer science or knowledge engineering sense of an explicit formal specification of the concepts in a domain (in this case, public health), their attributes and the relationships among them, which allows people (and computers) to share a common understanding of the structure of information. We chose this process, rather than a more traditional method for developing a classification, in recognition that a flexible, multidimensional classification structure would be needed in order to suit a variety of uses and user groups, and to exploit the near future capabilities of the Semantic Web. The Semantic Web is an initiative which aims to give meaning (semantics), in a manner understandable by machines, to the content of documents on the World Wide Web (and elsewhere) [33].

We used published methods for frame-based ontology development [34], and Protégé open-source, ontology-building software from Stanford University [35] as a development tool. After scanning available methods and software, we chose Protégé because it is freely available to everyone and does not require a commercial license, supports current Semantic Web standards such as the Resource Description Framework (RDF) [36] and Web Ontology Language (OWL) [37], and has an active community of interest, with strong representation from researchers in the health, biomedical and related sectors. Although Protégé provides support for the three types of OWL, we chose to develop our ontology in simpler CLIPS format in the first instance, as this is slightly easier to use, with a view to transforming it to OWL format at a later date.

The four steps in the ontology-building process were as follows:

1. Determine the domain and scope of the ontology.

2. Consider reusing existing ontologies.

3. Enumerate important terms in the ontology.

4. Define the classes and class hierarchy [34].

A reference group of public health experts (see Acknowledgements) drafted initial responses to steps 1 to 4. Definitions of public health and of core and essential public health functions, including those used by the WHO [13], the OECD system of health accounts [29], the Australian National Public Health Partnership [2], the US Department of Health and Human Services [10], the US Association of Schools of Public Health [38] and the Pan American Health Organisation [14], the UK Department of Health [11] and the Canadian National Advisory Committee on SARS and Public Health [16] (see Additional file 1) were reviewed for step 2.

We refined the classification through a series of consultations with public health experts and practitioners across Australia. The project consultation process sought to achieve agreement on a version of the classification that was 'good enough', recognising that the classification of public health is a complex and technically difficult problem, with no definitive formulation or solution. Early consultations were informal, and designed to seek the views of content experts in particular domains (e.g. environmental health, health promotion). Later, more formal consultations were organised through reference group members representing various jurisdictions.

Prior to each consultation meeting, we sent material introducing the classification project to participants. All consultations were conducted face to face. The number of participants varied from one or two, to larger groups of up to 15, and the duration varied from one to three hours. In each consultation, an introduction and background to the classification project were given with the aid of a slide presentation, after which an early version of the public health classification, rendered through a Web browser, was demonstrated. This was followed by a 'live' session using Protégé, which allowed participants to explore the structure of the classification, to suggest additions and changes, and to immediately see their effect on the overall classification.

Last, participants were asked to identify practical uses for a multidimensional public health classification. Following consultation meetings, the project reference group debated proposed changes to the classification before deciding to adopt or reject them. The Australian National Public Health Information Working Group – a committee with representation from all states and territories as well as relevant national bodies – discussed and provided feedback on an early draft version of the classification.

## Results

### Principles of development

Development of the public health classification was guided by the following principles:

1. The classification should be multidimensional.

2. A range of the most important dimensions need to be considered and developed concurrently.

3. Existing classification systems (including international and Australian standards) should be used wherever possible.

4. The classification should be inclusive and deliberately broad at the top levels. Specific boundaries and restrictions to the scope of the classification should be defined in practical applications, rather than be arbitrarily imposed during the development of the classification.

### The public health classification

**Figure 1:**
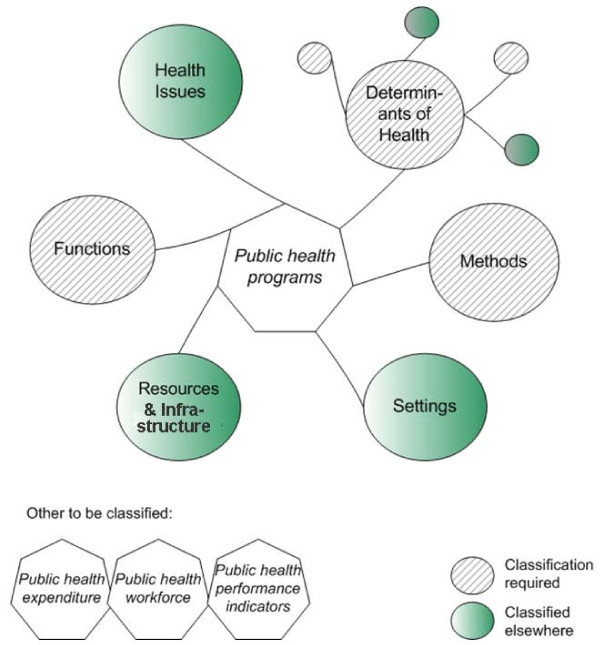
**A model of public health classification**. Source: Adapted from Gruszin S, Jorm L, Churches T, Straton J: *Public Health Classifications Project Phase One: Final Report: Report to the National Public Health Partnership Group*. Melbourne: National Public Health Partnership; 2005.

Version one of the public health classification consists of six top-level classes: (public health) 'Functions'; 'Health Issues'; 'Determinants of Health'; 'Settings'; 'Methods' (of intervention); and 'Resources and Infrastructure', which are shown as circles in Figure 1, together with a hierarchy of subclasses, and, at the lowest level, instances. Each subclass and instance should have a subsumption ("is-a") relationship with its parent class.

Existing classifications (such as the international classifications of diseases [25], functioning and disability [26], and external causes of injuries [27], can be used as subclasses of the classes 'Health Issues', 'Settings' and 'Resources and Infrastructure', while new subclass structures are proposed for the classes of public health 'Functions', 'Determinants of Health' and 'Methods' (see Figure 1).

The working definitions for the six top-level classes are shown in Table 1, and their immediate subclasses are given in Table 2.

**Table 1:** Classification of public health: six top-level classes and their working definitions

**Class**	**Working definition**
Functions	Public health functions. The purpose of public health interventions, actions, activities and programs.

Health Issues	Health, and well-being issues that affect health ('issues' includes: concerns, topics, problems). Health is defined (by the WHO) as 'a state of complete physical, mental and social well-being and not merely the absence of disease or infirmity'.

Determinants of Health	Factors that influence health status and determine health differentials or health inequalities. They include, for example, natural, biological factors, such as age, sex and ethnicity; behaviour and lifestyles, such as smoking, alcohol consumption, diet and physical activity; physical and social factors, including employment and education, housing quality, the workplace and the wider urban and rural environment; and access to health care [a].

Methods	The methods used by organised public health interventions (actions, activities, programs, services) to protect and promote health and prevent illness, injury and disability, that are designed to change population exposure, behavioural or health status.

Settings	Settings in which public health activities and interventions take place, institutional and social environments, partnerships, and locations (e.g. schools, local government, hospitals, workplaces).

Resources and Infrastructure	Resources and infrastructure, the means available for the operation of health systems, including human resources, facilities, equipment and supplies, financial funds and knowledge [b]. It includes both person-time and calendar time.

Source: Gruszin S, Jorm L, Churches T, Straton J: *Public Health Classifications Project Phase One: Final Report: Report to the National Public Health Partnership Group*. Melbourne: National Public Health Partnership; 2005.

[a] World Health Organisation: Health Impact Assessment (HIA): Glossary of terms used. Geneva; 2006.

[b] World Health Organization: Health Promotion Glossary. Geneva; 1998. [WHO/HPR/HEP/98.1]

**Table 2:** Classification of public health: top two levels of all classes

**Top-level class**	**Level 2 subclasses**		
**Functions**	Assess health of populations		
***Primary:***	Promote health and prevent disease, disability and injury		
	
	Protect from threats to health		

***Instrumental***	Ensure public health capability		
	
	Build the evidence base for public health		

**Health issues**	Health and well-being	Injury	
	
	Diseases and conditions	Disability and functioning	

**Determinants of Health**	Environmental	Socioeconomic	External causes of injury
	
	Person-level	Health system	

**Methods**	Advocacy and lobbying	Health impact assessment	Research and evaluation
	
	Communicable disease control specific	Immunisation	Road safety methods
	
	Community action	Infection control	Screening to detect disease/risk factors
	
	Community development	Legislation and regulation	Social action
	
	Counselling	Lifestyle advice	Social marketing
	
	Diagnosis	Management of biological risk	Training and workforce development methods
	
	Directed investment	Monitoring and surveillance	Treatment methods
	
	Environmental monitoring	Personal skills development	Urban planning methods
	
	Epidemiologic methods	Political action	Vector control methods
	
	Exercise of capabilities	Public policy development	Waste management methods
	
	Food safety methods	Radiation safety methods	Other methods of intervention
	
	Health education	Remediation of environment methods	

**Settings**	Educational settings	Home settings	Other settings
	
	Healthcare settings	Workplace settings	Includes LOCATIONS – classification of geographical areas (e.g. postcodes).
		
	Local government and communities settings	Transport settings	

**Resources and infrastructure**	Administrative infrastructure	Organisational systems	Technical infrastructure
	
	Funds	Partnerships	Time
	
	Information systems	Physical infrastructure	Workforce
	
	Legislative infrastructure	Policies	Workforce development capacity

Source: Gruszin S, Jorm L, Churches T, Straton J: *Public Health Classifications Project Phase One: Final Report: Report to the National Public Health Partnership Group*. Melbourne: National Public Health Partnership; 2005.

The 'Functions' class is currently the most highly developed. Both primary and instrumental functions were considered to be important in conceptualising public health (See Additional file 2). Primary functions are *ends *in themselves, while instrumental functions are *means *to those ends. Public health practitioners also described instrumental functions as 'supporting', 'underpinning' or 'cross-cutting' functions because all primary functions rely on them – they do not belong solely to any one of the primary functions.

While there was reasonable agreement among public health experts regarding the subclasses for the top-level classes 'Health Issues', 'Determinants of Health', and 'Settings', the remaining classes are in earlier stages of development.

The 'Methods' class was the subject of some disagreement, with some experts preferring to narrow its scope to those methods that are *peculiar *to – or only used by – public health (e.g. population-based epidemiology, health promotion, environmental risk assessment). Others favoured an inclusive approach that would capture *all methods *used by public health, including those that, while not specific to it, are employed by public health workers in the normal course of their work (e.g. administration, management, policy development). An inclusive approach was adopted, in line with development principle (4) above.

Public health experts expressed diametrically opposed views as to whether 'infrastructure' represented aggregates of 'resources', or whether 'resources' were in fact a subclass of 'infrastructure'. An inclusive approach was adopted, in line with development principle (4) above, with the relevant class termed 'Resources and Infrastructure', and its subclasses capturing both compound (e.g. administrative infrastructure, information systems) and unitary elements (e.g. funds, workforce).

Further work is needed to disentangle the mixture of partitive (holonym-meronym) and subsumption (hypernym-hyponym) relationships among the current subclasses of this top-level class.

A copy of the full report on this initial phase of the project has been included as an additional file to this paper (Additional file 3), together with copies of the underlying classification ontology as it stood at the time of writing. Two sets of ontology files are provided: a set of Protégé project files (Additional file 4), and a set of interlinked HTML files that can be explored using a Web browser (Additional file 5).

### Potential uses for the classification

The classification is used by assigning zero or more attributes chosen from each of the top-level class hierarchies to the "thing" being classified. A wide range of "things" can potentially be organised according to the classification, including (but not restricted to) public health policies, programs and interventions, the population groups they target, and their outcomes. Figure 1 depicts some examples of things that could be classified, shown as heptagons.

**Figure 2:**
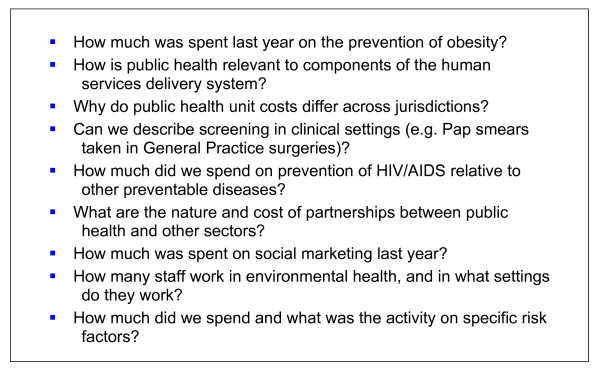
**A public health classification should help answer questions like...**. Source: Adapted from Gruszin S, Jorm L, Churches T, Straton J: *Public Health Classifications Project Phase One: Final Report: Report to the National Public Health Partnership Group*. Melbourne: National Public Health Partnership; 2005.

Many participants in the development process expressed a view that the classification would assist them in describing what public health is, and what its characteristics are. They also identified a range of questions that, currently, are difficult or impossible to answer, but which potentially could be answered if the multidimensional classification were used to facilitate aggregated reporting on public health activity. Examples of such questions are given in Figure 2.

**Figure 3:**
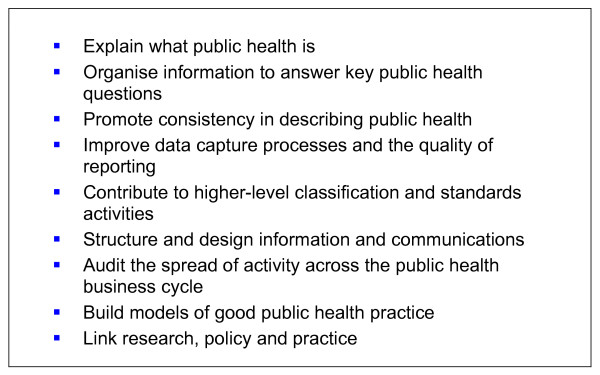
**Potential uses for a public health classification**. Source: Adapted from Gruszin S, Jorm L, Churches T, Straton J: *Public Health Classifications Project Phase One: Final Report: Report to the National Public Health Partnership Group*. Melbourne: National Public Health Partnership; 2005.

Participants also suggested a range of potential practical applications for the classification. Examples of these are given in Figure 3. A knowledge base to support communicable disease surveillance, also constructed using the Protégé software suite, has been described [39]; it should be possible to use the classification ontology to create analogous databases for other specific or general areas of public health practice.

## Discussion

The process of developing the classification brought to light several areas of basic disagreement among Australian public health practitioners regarding the nature of public health practice.

The inclusion, or otherwise, of preventive services delivered on a one-to-one basis to individuals was particularly contentious. Such preventive services include screening and detection, immunisation, and counselling and lifestyle advice to support healthy behaviour, as well as management (through lifestyle changes or pharmacological means) of disease risk factors such as high blood pressure and high cholesterol.

Many participants argued that those individual preventive services related to communicable disease (immunisation, contact tracing and treatment for sexually transmitted infections) are public health activities because they help to protect the health of the whole population, through herd immunity and controlling the spread of infection. Others felt that immunisation is only a legitimate part of public health activity when it is delivered as part of an organised government-funded program, such as through local government or school health services.

Most public health practitioners agreed that early detection of disease through screening is a public health activity when it is delivered through an organised government-funded program (such as national breast and cervical cancer screening programs). Less clear was whether screening that is not part of an organised program, such as opportunistic bone density screening for osteoporosis, should be seen as part of public health.

There was substantial disagreement among public health practitioners regarding the inclusion, or otherwise, of activities relating to prevention and management of non-communicable disease through individual counselling or other interventions directed at lifestyle risk factors (smoking, poor nutrition, risky alcohol use and lack of physical activity), and the early detection and management of biological risk factors such as high blood pressure and high cholesterol. Many contended that the diagnosis of a pathological condition (such as high blood pressure) or disease marked the boundary of public health practice. Others regarded this boundary as spurious, because the pathophysiological processes which underlie the development of chronic disease are continuous, and because interventions such as anti-hypertensive and cholesterol-lowering drugs, or even the 'Polypill' [40], may have dramatic benefits in terms of morbidity and mortality at the population level.

By adopting an inclusive approach, the public health classification allows decisions about boundaries, inclusions and exclusions, to be made at the level of individual applications of the classification. This is especially useful for those boundary issues – such as where public health ends and clinical practice begins – about which opinion may evolve with knowledge about preventive interventions, and how and by whom they are best delivered.

The issue of whether a public health classification should be restricted to a domain solely within the health sector or whether it should it include the health-related activities of other sectors was also frequently raised. Most public health practitioners agreed, when pressed, that accounting for public health should include the activities of, and investments by, the non-health portfolios (such as education and transport) of national and state governments, as well as the relevant activities of local governments and non-government organisations (NGOs). This is consistent with the definition of public health used by the US Institute of Medicine as: "what we, as a society, do collectively to assure the conditions for people to be healthy" [9].

However, in practice there are major difficulties in capturing information on public health-related activities and expenditure by non-health sectors. In Australia, current public health expenditure reporting is limited to the health portfolio expenditures of the state and national governments [1]. One view was that the activities of non-health sectors should only be considered 'in scope' when public health is their primary purpose (e.g. immunisation organised by local government). The 'boundary' issues we encountered reflect the way that public health activity is conceptualised, and organised, in Australia. Similar exercises conducted in other countries would doubtless highlight different issues. For example, public health services in the US (which has a strongly privatised approach to health care) are seen as having a major role in filling 'gaps' in health service provision (such as maternal and child health services) for those without access to health insurance, as well as in evaluating the accessibility and quality of personal health care services [10]. In several European countries, provision of many public health services is fully devolved to the level of local municipalities, which also have responsibility for issues such as air and water quality, noise diminution and waste management. It is likely that the organisation of services in these countries influences the way their public health practitioners conceptualise the boundaries between primary health care, public health and environmental protection.

A comparison of the published public health functions of other nations (see Additional file 1) shows that some (e.g. those of Canada [16] and the Americas [14,15]) are limited to primary functions, while others include both primary and instrumental functions. In the UK [11], both primary (e.g. health promotion and disease prevention programs) and instrumental (e.g. development and maintenance of a public health workforce) functions are prominent. Our classification captures the majority of functions that other nations have described, including the (instrumental) 'partnership' and 'research' functions that are present in both the UK core functions [11] and the 10 essential public health services of the USA [10].

A 'quality assurance' function does not currently feature in our classification, although it is specified in the published functions for public health in the USA [10], the Americas [14,15], the UK [11] and in WHO's most recent work [13,15].

Public health practitioners and experts in Australia at no stage suggested that such a function was a critical part of their work. It is possible that their views may subsequently have changed, particularly in light of several recent scandals [41-43] relating to the safety and quality of the care provided in Australian public hospitals.

The approach we adopted in developing the classification should maximise its flexibility for application in other settings. The ontology-building process offered particular advantages in dealing with divergent (and often strongly held) views regarding what was and was not 'in scope'. Although defining and specifying classes was central to the process, the emphasis was on modelling the relationships among classes, rather than on the within-class hierarchies. We were able to adopt an inclusive approach, leaving scope for decisions about rules and exclusions to be made at the level of specific practical applications of the classification. It is to be hoped that such practical applications will make the areas of contention explicit, encourage debate, and offer a way to move towards a common language to describe public health activity in Australia and elsewhere.

## Conclusion

The public health classification is an initial attempt to describe the important dimensions of public health activity. It will facilitate the organisation of information about public health activity so that it can be used to address questions relating to any of these dimensions, either singly or in combination. The authors encourage readers to use the classification, and to suggest further refinements. It is our intention to further refine and extend several of the class hierarchies, and to convert the ontology into RDF and OWL format to make it suitable for use in Semantic Web applications. We welcome potential collaborators in this endeavour.

## Abbreviations

HIV/AIDS: Human Immunodeficiency Virus/Acquired ImmunoDeficiency Syndrome; OECD: Organisation for Economic Co-operation and Development; UK: United Kingdom; US: United States of America; WHO: World Health Organization; RDF: Resource Description Framework; OWL: Web Ontology Language (sic).

## Competing interests

The authors declare that they have no competing interests.

## Authors' contributions

LJ compiled the argument and drafted the background, discussion and conclusions of the manuscript. SG reported the results, contributed to the background and discussion, and prepared the figures and tables. TC informed the directions of the project, provided key conceptual and practical input to its results, and reviewed and edited the manuscript. All authors worked on the project, and have co-authored, with Judy Straton (see Acknowledgements), the report of phase one of the project. All authors read and approved the final manuscript.

## Supplementary Material

Additional file 1 **Comparison of published public health functions and roles**. Table presenting public health roles and functions published in international reports. Click here for file

Additional file 2 **Classification of public health: detail of the functions class**. Table presenting the "Functions" class of the public health classification, and its subclasses. Click here for file

Additional file 3 **Gruszin S, Jorm L, Churches T, Straton J: *Public Health Classifications Project Phase One: Final Report: Report to the National Public Health Partnership Group*. Melbourne: National Public Health Partnership; 2005**. Full report of Phase One of the Australian Public Health Classifications Project. Click here for file

Additional file 4 **Public Health ontology (PHont) Version 1.0**. Protégé version 3.1 project files for Public Health Ontology (PHont) Version 1.0. Click here for file

Additional file 5 **Public Health ontology (PHont) Version 1.0**. HTML web page version of Public Health Ontology (PHont) Version 1.0 Click here for file
