# Supplementary material for: A multidimensional classification of public health activity in Australia
Source: Aust New Zealand Health Policy. 2009 Apr 9;6:9. doi: 10.1186/1743-8462-6-9 (PMC2674448; doi:10.1186/1743-8462-6-9)
Supplement: Additional file 1 — Comparison of published public health functions and roles. Table presenting public health roles and functions published in international reports. [file 1743-8462-6-9-S1.pdf]

Additional file 1: Comparison of published public health functions and roles

Canada 2003 (a)	UK 2003 (b)	WHO 2003 (c)	The Americas 2002 (d)	OECD 2000 (e)	Australia 2000 (f)	USA 1994 (g), 1988 (h)
**Essential Public Health Functions:**<br>▪ Health protection<br>▪ Health surveillance<br>▪ Disease and injury prevention<br>▪ Population health assess-ment<br>▪ Health promotion<br>▪ Disaster response	**Public Health Core Functions**:<br>▪ Health surveillance, monitoring and analysis<br>▪ Establishing, designing and managing health promotion and disease prevention programmes<br>▪ Enabling and empowering communities and citizens to promote health and reduce inequalities<br>▪ Creating and sustaining cross-governmental and inter-sectoral partnerships to improve health and reduce inequalities<br>▪ Ensuring compliance with regulations and laws to protect and promote health<br>▪ Developing and maintaining a well-educated and trained, multidisciplinary public health workforce<br>▪ Ensuring the effective performance of NHS services to meet goals in improving health, preventing disease and reducing inequalities<br>▪ Research, development, evaluation and innovation and quality assuring the public health function.	**Essential Public Health Functions:**<br>1 Health situation monitoring & analysis<br>2 Epidemiological surveillance/ disease prevention & control<br>3 Development of policies & planning in public health<br>4 Strategic management of health systems & services for population health gain.<br>5 Regulation and enforcement to protect public health.<br>6 Human resources development & planning in public health.<br>7 Health promotion, social participation & empowerment<br>8 Ensuring the quality of personal & population-based health services.<br>9 Research, development & implementation of innovative public health solutions.	**Public Health Roles:**<br>▪ Prevents epidemics and the spread of disease<br>▪ Protects against environmental hazards<br>▪ Prevents injuries<br>▪ Promotes and encourages healthy behaviors<br>▪ Responds to disasters and assists communities in recovery<br>▪ Assures the quality and accessibility of health services<br>**Essential Public Health Functions:**<br>1 Health situation monitoring & analysis<br>2 Public health surveillance, research, & control of risks & damages in public health<br>3 Health promotion<br>4 Social participation & empowerment of citizens in health<br>5 Development of policy & planning to support individual & collective efforts in public health & the steering role of the National Health Authority<br>6 Public health regulation & enforcement<br>7 Evaluation & promotion of equitable access to necessary health services	**Prevention and public health services:**<br>▪ Maternal and child health; family planning and counselling<br>▪ School health services<br>▪ Prevention of communicable diseases<br>▪ Prevention of non-communicable diseases<br>▪ Occupational health care<br>▪ All other miscellaneous public health services<br>**Health-related functions** include:<br>▪ Food, hygiene & drinking water control; and<br>▪ Environmental health).	**Public Health Core Functions**:<br>1 Assess, analyse & communicate population health needs & community expectations<br>2 Prevent & control communicable & non-communicable diseases & injuries through risk factor reduction, education, screening, immunisation & other interventions<br>3 Promote & support healthy lifestyles & behaviours through action with individuals, families, communities & wider society<br>4 Promote, develop & support healthy public policy, including legislation, regulation & fiscal measures<br>5 Plan, fund, manage & evaluate health gain & capacity building pro-grammes designed to achieve measurable imp-rovements in health status, & to strengthen skills, competencies, systems & infrastructure<br>6 Strengthen communities & build social capital through consultation, participation & empowerment<br>7 Promote, develop, support & initiate actions which ensure safe & healthy environments<br>8 Promote, develop & support healthy growth & development throughout all life stages<br>9 Promote, develop & support actions to improve the health status of Aboriginal & Torres Strait Islander people & other vulnerable groups	**Essential Public Health Services (1995):**<br>1 Monitor health status to identify community health problems.<br>2 Diagnose & investigate health problems & health hazards in the community.<br>3 Inform, educate, & empower people about health issues.<br>4 Mobilize community partnerships to identify & solve health problems.<br>5 Develop policies & plans that support individual & community health efforts.<br>6 Enforce laws & regulations that protect health & ensure safety.<br>7 Link people to needed personal health services & assure the provision of health care when otherwise unavailable.<br>8 Assure a competent public health & personal health workforce.<br>9 Evaluate effectiveness, accessibility, & quality of personal & population-based health services.<br>10 Research for new in-sights & innovative solutions to health problems.<br>**Public Health Core Functions (1988):**<br>▪ *Assessment* - of a community's health and its resources.<br>▪ *Policy development* - to promote health and solve health problems.<br>▪ *Assurance* - that access to health care, promotion, and prevention services are available.

Sources:
(a) National Advisory Committee on SARS and Public Health: Learning from SARS: Renewal of public health in Canada. Ottawa: Health Canada; 2003 [16].
(b) Chief Medical Officer, United Kingdom: Public health in England. London: UK Dept of Health; 2003 [11].
(c) World Health Organization (WHO), Regional Office for the Western Pacific: Essential public health functions: A three-country study in the Western Pacific Region. Manila, Philippines; 2003 [13].
(d) Pan American Health Organization: Public Health in the Americas: conceptual renewal, performance assessment, and bases for action. Washington, DC; 2002 [14].
(e) Ramagem C, Ruales J: The essential public health functions as a strategy for improving overall health systems performance: trends and challenges since the Public Health in the Americas Initiative, 2000-2007. Washington, DC: Pan American Health Organization/World Health Organization; 2008 [15].
(f) Organisation for Economic Co-operation and Development (OECD): International Classification of Health Accounts. Geneva; 2000 [28].
(g) National Public Health Partnership: Public health practice in Australia today – a statement of core functions. Melbourne; 2000 [2].
(h) Public Health Functions Steering Committee, US Department of Health and Human Services: Public health in America. Washington DC: US Department of Health and Human Services; 1994 [10].
(i) Institute of Medicine: The future of public health. Washington, DC: National Academy Press; 1988 [9].
