# Supplementary material for: A multidimensional classification of public health activity in Australia
Source: Aust New Zealand Health Policy. 2009 Apr 9;6:9. doi: 10.1186/1743-8462-6-9 (PMC2674448; doi:10.1186/1743-8462-6-9)
Supplement: Additional file 2 — Classification of public health: detail of the functions class. Table presenting the "Functions" class of the public health classification, and its subclasses. [file 1743-8462-6-9-S2.pdf]

Additional file 2: Classification of public health: detail of the functions class

Top-level	Level 2		Level 3	
	**Class**	**Definition**	**Class**	**Definition**
**Functions: Primary functions (ends)**	Assess health of populations	Monitor and analyse health and determinants of health in populations, assess the impacts of policies, interventions, and environmental exposures.	Monitor health	Monitor and analyse levels of health and its determinants in populations to identify and predict trends and emerging issues
			Evaluate health risks and benefits	Evaluate adverse and beneficial effects related to health and social policies and interventions, and environmental exposures.
			Assess health inequalities	Assess inequalities in health (level and distribution) and health gain to target interventions to improve the health of the worst-off sub-populations.
	Protect from threats to health	Protect from, and prevent, external threats to public health.	Prepare for threats to health	Minimise or reduce the severity of risks to health (includes setting and monitoring of standards for e.g. food, air and water quality and other potential hazards, also harm minimisation measures).
			Respond to threats to health	Respond to threats to health (including communicable diseases, environmental hazards, bio-terrorism and other disasters).
			Control and mitigate risks to health	Identify and prepare for potential threats to health (including communicable diseases, environmental hazards, bio-terrorism and new patterns of exposures e.g. arising from ecological change).
	Promote health and prevent disease, disability and injury	Promote health and wellbeing, prevent the occurrence of disease, disability and injury; and detect disease in its early stages, through organised efforts that target populations.	Promote health and wellbeing	Promote better health and well-being as it affects health (e.g. community development and community empowerment initiatives clearly differentiated from 'Prevent the occurrence of...').
			Prevent the occurrence of disease, disability and injury	Prevent the initial occurrence of disease, disability and injury (e.g. population-level campaigns to promote physical activity, tobacco control, seat belt legislation).
			Detect disease, disability or injury in its early stages	Detect disease, disability and risk of injury early and initiate prompt management or response (e.g. screening for cancers, newborn hearing screening).
**Instrumental functions (means to achieve ends)**	Ensure public health capability	Ensure adequate public health capacity and responsiveness by maintaining and developing the public health workforce and infrastructure, and building partnerships with other sectors of society.	Develop and maintain the public health workforce	Train, maintain and develop the public health workforce.
			Develop and maintain public health infrastructure	Build, maintain and develop public health infrastructure, including physical, organisational, legislative, communication and informational, logistical, and other systems forming the public health infrastructure.
			Build public health partnerships	Build and maintain public health partnerships with other sectors and the community, to work together on shared issues and undertake actions towards agreed targets.
	Build the evidence base for public health	Increase and enhance the bodies of knowledge and evidence that inform public health practice (e.g. research, research synthesis, evaluation).	Conduct public health research	Conduct public health research.
			Evaluate public health interventions	Evaluate public health interventions.
