# Supplementary material for: A multidimensional classification of public health activity in Australia
Source: Aust New Zealand Health Policy. 2009 Apr 9;6:9. doi: 10.1186/1743-8462-6-9 (PMC2674448; doi:10.1186/1743-8462-6-9)
Supplement: Additional file 3 — Gruszin S, Jorm L, Churches T, Straton J: Public Health Classifications Project Phase One: Final Report: Report to the National Public Health Partnership Group. Melbourne: National Public Health Partnership; 2005. Full report of Phase One of the Australian Public Health Classifications Project. [file 1743-8462-6-9-S3.pdf]

Public Health Classifications Project

Phase One: Final Report

Report to the National Public Health Partnership Group

December 2005

National Public Health Partnership

Report prepared for the National Public Health Information Working Group of the National Public Health Partnership.

Report prepared by Su Gruszin, Louisa Jorm, Tim Churches and Judy Straton.

Su Gruszin is the Project Officer for the Public Health Classifications Project, and a Research Fellow at the Public Health Information Development Unit, The University of Adelaide.

Louisa Jorm is the Project Manager of the Public Health Classifications Project and Director of the Centre for Epidemiology and Research, NSW Department of Health.

Tim Churches, NSW Health, is the public health content and ontology technical advisor assisting the project.

Judy Straton is a member of the Project Reference Group, and the Director of the Child and Community Health Directorate, WA Department of Health.

Acknowledgements

Thank you to the many people who contributed ideas as part of the consultations. See *Appendix B* for a full list of persons consulted.

The **Public Health Classifications Project Reference Group** assisted with the production of this Report.

Chair: Louisa Jorm, NSW Dept of Health (and National Public Health Partnership Group representative on the National Health Performance Committee).

Members:

Michael Ackland (Department of Human Services, Victoria)

Andrea Casasola (Queensland Health)

Jenny Cleary, Pam Gollow (Department of Health and Community Services, NT)

Charles Guest (ACT Health)

Paul Jelfs (SA Department of Health)

Paul Magnus and Catherine Sykes, Robert Van der Hoek (Australian Institute of Health and Welfare)

Dean Martin (Australian Bureau of Statistics)

Karen Roger (National Public Health Partnership Secretariat)

Colin Sindall (Population Health Division, Australian Government Department of Health and Ageing)

Judy Straton (Department of Health WA)

Tony Woollacott (Expenditure Project representative, SA Department of Health; Chair, National Public Health Expenditure Project Technical Advisory Group)

© National Public Health Partnership, December 2005

Contents

Executive summary **1**

1 Introduction **5**

2 Methods **9**

3 Results **11**

3.1 Scope and domain *11*
3.1.1 Definition 11
3.1.2 Boundary issues 11
3.1.3 Potential uses 13
3.1.4 Who will use and maintain the classification? 16
3.1.5 Principles of development 16
3.1.6 Issues for further consideration 16

3.2 Version one of a public health classification *20*
3.2.1 Top-level classes and working definitions 20
3.2.2 Public health functions 22
3.2.3 Other top-level classes 29
3.2.4 Using the public health classification 30
3.2.5 Other classes considered 33
3.2.6 Issues for further consideration 34

4 Next steps and recommendations **35**

Glossary **37**

References and sources **45**

Acronyms and abbreviations **50**

Appendices **51**

Appendix A: Project inception and terms of reference *51*

Appendix B: Acknowledgement of contributors *53*

Appendix C: Defining public health *65*

Appendix D: Contextual drivers and technical context *67*

Appendix E: Names: population health and public health *70*

Boxes

Box 1 Potential uses for a public health classification 13
Box 2 Issues of definition for further consideration 19
Box 3 Classification of public health: other issues for further consideration 34

Figures

Figure 1 Classification of public health: top-level classes 2
Figure 2 A model of public health classification 3
Figure 3 Conceptions of public health over time (B-D) 17
Figure 4 Overlapping concepts relating to public health 18
Figure 5 Classification of public health: top-level classes 21
Figure 6 Other national public health functions 24
Figure 7 A model of public health classification 30
Figure 8 Detail of a classified public health activity 31
Figure 9 Detail of a classified public health policy 32
Figure 10 Using the UK Public Health Information Tagging Standard 63
Figure 11 UK National Public Health Language including top-level classes 64
Figure 12 Multiple inheritance of classes in an ontology 69

Tables

Table 1 An overview of public health functions 3
Table 2 Classification of public health: top-level classes and working definitions ... 22
Table 3 Classification of public health: overview of the functions class 23
Table 4 Classification of public health: working definitions of functions subclasses 26
Table 5 Functional equivalents of the public health core functions 27
Table 6 Classification of public health: top levels of all classes 29
Table 7 Sample of departmental names in jurisdictions 70
Table 8 Sample of school names in universities 70

Executive summary

The objective of the Public Health Classifications Project is to 'develop and endorse a higher-level classification that captures the breadth and scope of public health activity and provides a unified framework for multiple uses'. Such a unified framework will assist in improving the quality and consistency of reported information on public health activity, performance, investment and expenditure. The National Public Health Partnership funded the project in response to recommendations from the 2002 Public Health Performance Project.[1]

During the early scoping stages of the Public Health Classifications Project, it became apparent that a simple, one-dimensional classification system for public health could not satisfy the needs, or reflect the diverse world-views, of its disparate potential users. To provide a single '*unified* framework' for *multiple* public health uses, a multi-dimensional classification was needed.

In the domain of public health, a flexible and inclusive approach offers particular advantages, because there are divergent (and strongly held) views regarding what is and is not 'in scope'. By making such issues explicit, the process of developing a public health classification potentially offers a way to move towards a common language to describe public health activity in Australia, and to develop a practical tool that will improve data collection processes and the utility of public health information.

This report is the output of phase one of the Public Health Classifications Project. It introduces the concept of a multi-dimensional public health classification and describes the challenges encountered in developing it. The report presents version one of a classification of public health, outlines some potential practical applications, and proposes the next steps for phase two of the project.

Methods

A Reference Group (see acknowledgements in *Appendix B*) oversaw phase one of the Public Health Classifications Project and provided ongoing expert advice and comment.

The project used a formal methodology and supporting software.[2] A review of current public health definitions, concepts and relevant classifications was used to develop the scope, domain, and initial multi-dimensional structure. These were considered in a series of consultations with public health experts across Australia (see list of those consulted in *Appendix B*). Consultations involved both one-on-one meetings and larger group sessions. Experts identified important omissions, fine-tuned concepts, and nominated practical uses for the public health classification.

---

[1] Owen & Jorm 2002.

[2] *Ontology development 101* (see Noy & McGuinness 2001), and *Protégé* open source, ontology-building software from Stanford University.

Domain and scope

The definition of public health adopted was as follows:

> Public health is the organised response by society to protect and promote health, and to prevent illness, injury and disability. The starting point for identifying public health issues, problems and priorities, and for designing and implementing interventions, is the population as a whole, or population sub-groups.[3]

The boundary between public health and clinical practice came up repeatedly as an issue in discussions about the scope of public health, with particular debate about whether preventive services delivered on a one-to-one basis to individuals should be considered in scope. Many agreed that immunisation was in scope because it is an activity that is 'organised' at a population level with benefits for both populations and individuals. More contentious, however, was the possible inclusion of interventions that are designed to prevent and manage chronic diseases, and that are delivered to individuals in primary care settings.

Whether or not public health is a domain solely within health or whether it includes activities in other sectors (e.g. education, transport, local government) was also debated, particularly where the public health impact of the activities in these other sectors is incidental, rather than the primary purpose of the activity.

The general approach adopted in producing the classification was to be inclusive, and to allow decisions about specific exclusions to be made at the later stages when developing individual applications and uses of the classification.

Version one of a classification of public health

The broad structure of version one of a classification of public health consists of six top-level classes as shown in *Figure 1*.

There was consensus among the public health experts consulted, that a public health classification should be multi-dimensional, and there was broad agreement on the top-level classes that should be included.

Figure 1 Classification of public health: top-level classes

FUNCTIONS
HEALTH ISSUES
DETERMINANTS OF HEALTH
METHODS
SETTINGS
RESOURCES & INFRASTRUCTURE

Public health functions are defined as the purpose of public health interventions, actions, activities and programs. The 'functions' class was developed from the National Public Health Partnership *public health core functions*[4] and includes both primary and instrumental functions (shown in *Table 1*).

---

[3] National Public Health Partnership 1998.

[4] National Public Health Partnership 1998.

Table 1 An overview of public health functions

Top-level class	Level 2 subclasses	Level 3 subclasses
**Functions:** **Primary functions (ends)**	Assess health of populations	Monitor health
		Evaluate health risks and benefits
		Assess health inequalities
	Protect from threats to health	Prepare for threats to health
		Respond to threats to health
		Control and mitigate risks to health
	Promote health and prevent disease, disability and injury	Promote health and wellbeing
		Prevent the occurrence of disease, disability and injury
		Detect disease, disability or injury in its early stages
**Instrumental functions (the means to achieve the primary functions)**	Ensure public health capability	Develop and maintain the public health workforce
		Develop and maintain public health infrastructure
		Build public health partnerships
	Build the evidence base for public health	Conduct public health research
		Evaluate public health interventions

There was reasonable agreement regarding the top levels of the 'health issues' class (although its name was the subject of some debate), and the 'determinants of health' and 'settings' classes. The remaining classes are less well developed and have had limited testing through consultations.

As shown in *Figure 2*, existing classifications (such as the international classific-ations of diseases, functioning and disability, and external causes of injuries; and various Australian standards) are available to classify the classes of 'health issues', 'settings' and 'resources'. The National Public Health Information Working Group has determined that the further development of classifications for the top-level classes of 'functions', 'determinants of health' and 'methods' are required and a priority.

Figure 2 A model of public health classification

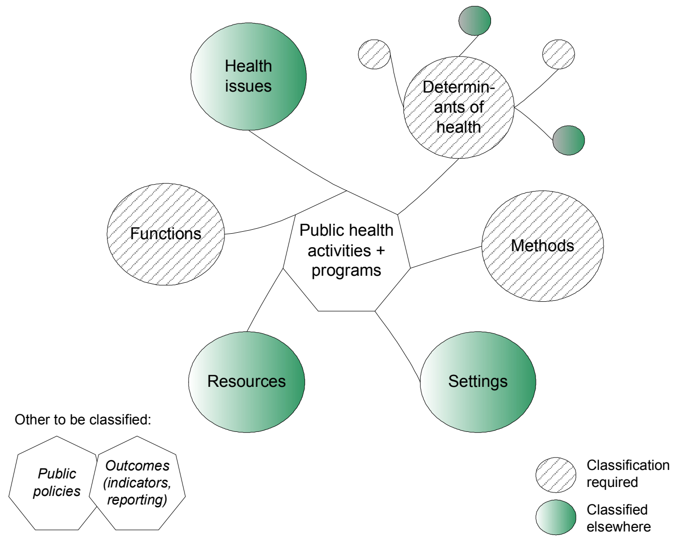

Potential uses

A public health classification should facilitate the organisation of information to answer key public health questions that cannot currently be answered, such as 'How much was spent last year on the prevention of obesity?' It should assist in describing what public health is, and what its characteristics are, through the development of classes that capture the functions of public health, issues of public health concern (including determinants of health), the settings in which public health operates, the population groups targeted, resources available and so on.

Potential practical applications for a public health classification include:

- Explaining what public health is;
- Organising information to answer key public health questions;
- Promoting consistency in describing public health;
- Improving data capture processes and the quality of reporting;
- Contributing to higher-level classification and standards activities;
- Lending structure to the design of public health information and communication systems;
- Auditing the spread of activity across the public health business cycle;
- Building models of good public health practice; and
- Linking research, policy and practice.

Who will use and maintain the classification?

Potential users of a public health classification include the various levels of government and other sectors that have an investment in public health, academics and students, researchers, evaluators, those involved in policy formulation, and anyone with an interest in public health.

The Australian Institute of Health and Welfare has indicated an interest in the longer-term development and maintenance of a public health classification.

Proposed next steps in the development of the classification

It is recommended that phase two of the Public Health Classifications Project should:

- Focus on further developing the classes of public health 'functions', 'determinants of health' and 'methods';
- Develop and release a web-based version of the public health classification with facilities for eliciting structured feedback and managing contributions to the further development and refinement of the classification;
- Develop a plan for ongoing development, support and governance of the public health classification;
- Further specify links or relations between the public health classification and relevant existing classifications and standards (with due regard for intellectual property rights); and
- Investigate inclusion of the public health classification in the Australian Family of Classifications.

1 Introduction

A classification is an 'arrangement of concepts into classes and their subdivisions to express the semantic relations between them'.[5] The essential characteristic of a classification is aggregation according to logical rules. Standardised, shared classifications are needed if we want to compare information about entities and discern their similarities and differences.

The objective of the Public Health Classifications Project is to 'develop and endorse a higher-level classification that captures the breadth and scope of public health activity and provides a unified framework for multiple uses'. Such a unified framework will assist in improving the quality and consistency of reported information on public health activity, performance, expenditure and investment. The National Public Health Partnership funded the Public Health Classifications Project in response to recommendations from the 2002 Public Health Performance Project.[6]

This report is the output of phase one of the Public Health Classifications Project. It introduces the concept of a multi-dimensional public health classification and describes the challenges encountered in its development. It presents version one of a public health classification, outlines some potential practical applications, and proposes the next steps for phase two of the project.

The public health sector in Australia

The National Public Health Partnership defines public health as:

> the organised response by society to protect and promote health, and to prevent illness, injury and disability. The starting point for identifying public health issues, problems and priorities, and for designing and implementing interventions, is the population as a whole, or population sub-groups.[7]

As a sector, public health is largely funded by government.[8] In Australia the Australian Government is the major source of public health funding, while state and territory governments mostly apply the funds.[9]

The public health workforce is as diverse as are its employers: there is no single or all encompassing occupation or industry group. The workforce is shaped like a pyramid with a small percentage of dedicated public health specialists, while the majority are

---

[5] ISO 1988.

[6] Owen & Jorm 2002. See *Appendix A* for additional information on the Project inception.

[7] NPHP 1998.

[8] Government funding is an important tracer for public health activity (Khalegian & Das Gupta 2004).

[9] Commonwealth provides 56% of funds, state and territory governments 44%. Commonwealth applies 30% of funds, state and territory governments 70%. Non-health funding (e.g. departments of transport for road safety/injury prevention, local government for a range of public health services) are not included (AIHW 2004b).

'general health and associated workers' who carry out aspects of public health functions on either a regular or occasional basis.[10]

Some public health activities are carried out in sectors outside of health (e.g. local government, non-government organisations (NGOs), other government departments and agencies, including planning and environmental protection agencies). Some 'classic' public health functions are outsourced and funded well away from health and human services portfolios (e.g. sewage disposal, provision of safe potable water).

Public health activity is costed at the program level,[11] and effectiveness and other measures are estimated at the aggregate level as theoretical constructs (e.g. population health status, potentially avoidable mortality). It is difficult to tell when public health effort and investment is effective, even over long periods of time; the small amount of work to this end is bedevilled by the poor quality of available data, the complexity of costing public health activity,[12] and lack of agreement about what should be included.[13] Costs to society when public health fails (e.g. cryptosporidium outbreak response, effect of SARS panic) may be easier to estimate.

Available expenditure estimates suggest that there are relatively high overheads or indirect costs for public health programs and activities (e.g. design and coordination costs, costs of administering and managing complex operations).[14] Public health tends to exhibit large economies of scale and to be relatively insensitive to population size; hence unit costs may be lower in states with larger populations to absorb the fixed costs of overheads.[15]

Why do we need a public health classification?

The National Public Health Partnership funded the Public Health Classifications Project in response to the 2002 Public Health Performance Project,[16] which recommended that the National Public Health Information Working Group undertake the development of a classification system for public health that could be used to further develop the categories used by the National Public Health Expenditure Project and

---

[10] Employers include Australian, state and territory, and local governments; NGOs, Aboriginal Community Controlled Health Organisations, community services, environmental protection services, health promotion foundations, private sector organisations (e.g. pharmaceutical companies, pathology laboratories) (Riddout et al. 2002: 8).

[11] Even when program categories are artificially created, for example, state reporting against 'activity categories' in public health expenditure reporting (see AIHW 2004b).

[12] Bennett 2003.

[13] Abelson analysed the epidemiological and economic effects of five public health programs over decades (including programs to reduce: tobacco consumption, coronary heart disease - which some would dispute as a public health program - and road trauma), estimating costs of investment in public health interventions and benefits in terms of total return to society, and, savings to government. The 'net present value' to government of road safety programs and programs to reduce coronary heart disease was estimated as negative (expenditure greater than savings); while the benefit of immunisation for Haemophilus influenzae B disease was estimated at a 'marginal $10 million' (Abelson et al. 2003: 4).

[14] AIHW 2004b, nine public health programs in all jurisdictions.

[15] Riddout et al. 2001.

[16] Owen & Jorm 2002.

performance monitoring by the National Public Health Partnership, and to inform a future review of the core functions for public health.

The Public Health Performance Project used the *public health core functions* that were endorsed by the National Public Health Partnership in 2000, to develop performance indicators for public health.[17] These core functions differ from the categories used for national public health expenditure reporting[18], resulting in difficulties in aligning data on performance with that on expenditure. More recently, a national report of health expenditure by disease groupings *excluded* expenditure on public health because this was not available 'by disease'[19]—further highlighting the inadequacy of current systems for capturing information about public health activities.

The objective adopted by the National Public Health Partnership for the Public Health Classifications Project was to 'develop and endorse a higher-level classification that captures the breadth and scope of public health activity and provides a unified framework for multiple uses'.

The project objective is to develop and endorse a higher-level classification that captures the breadth and scope of public health activity and provides a unified framework for multiple uses.

During the early scoping stages of the project, it became apparent that a one-dimensional classification of public health might look very different, depending on its intended use, and user group. A simple, one-dimensional classification of public health could not satisfy all the needs, or gel with the diverse world-views, of its disparate potential users. To provide a '*unified* framework for *multiple* uses', a multi-dimensional public health classification with explicit modeling of the relationships among dimensions is needed, rather than a single, mutually exclusive, hierarchy of categories.[20]

This project used an ontology-building process to develop the public health classification. An ontology is an explicit formal specification of the concepts in a domain (in this case, public health), their attributes and the relations among them, which allows people to share a common understanding of the structure of information.[21]

A multi-dimensional public health classification allows structure to be imposed on diverse material along different—but equally meaningful—dimensions, based on the way that public health experts and practitioners think about public health, and the ways in which they describe or classify it, or aspects of it, depending on their purpose.

In the domain of public health, this flexible and inclusive approach offers particular advantages, because there are divergent (and strongly held) views regarding what is

[17] Owen & Jorm 2002: 8.

[18] NPHP 1998, NHPC 2004, AIHW 2004b.

[19] AIHW 2004c.

[20] In practice, most classifications of complex domains are multi-dimensional, either implicitly so, or explicitly constructed as such. An example in the health field is the *International Classification of Diseases* (WHO 1992-94), although the relationships among the dimensions are not all set out overtly.

[21] Noy & McGuinness 2001.

and what is not 'in scope'. By making such issues explicit, the process of developing a classification offers a way to move towards a common language to describe public health activity in Australia, and to develop a tool to improve data collection processes and the consistency of information about public health activity, performance, expenditure, effectiveness and returns on investment.

2 Methods

A Reference Group (see acknowledgements in *Appendix B*) oversaw phase one of the Public Health Classifications Project and provided ongoing expert advice and comment.

The project used the *Ontology development 101* methodology[22] and the open source *Protégé* ontology-building software (from Stanford University[23]) as the development tools. *Ontology development 101* and *Protégé* were selected after a scan of available methods and software, because they were considered to be the most useful tools for the work, are openly available (i.e. do not require a commercial license), provide support for emerging Semantic Web standards, and have active communities of interest with strong representation from researchers and knowledge workers in health, biomedical and other related fields.

The steps followed in the public health classification building process were:

- Step 1 Determine the domain and scope of the classification:
  - What is the domain that the classification will cover?
  - For what are we going to use the classification?
  - For what types of questions should the information in the classification provide answers?
  - Who will use and maintain the classification?
- Step 2 Consider reusing existing classifications.
- Step 3 Enumerate important terms in the classification.
- Step 4 Define the classes and class hierarchy.[24]

Public health definitions and relevant classification systems, especially functional classifications, were reviewed for Step 2 and are available from the project.

The Project Reference Group workshopped the preliminary material and drafted initial responses to Steps 1 to 4. Consultations with public health content experts in a sample of jurisdictions considered the class hierarchy and its top levels, and identified important omissions. They also identified further practical uses of the classification.

Initial consultations were held in NSW from October 2004. Formal consultations were held in Brisbane, Melbourne, Canberra and Perth. Early consultations were informal, designed to seek the views of content experts on particular components (e.g. environmental health, health promotion). Later consultations were organised through Reference Group members representing various jurisdictions. Prepared material introducing the project was sent out to participants prior to each consultation. All consultations were face to face. The number of participants varied from one or two, to larger groups of up to fifteen, and the duration varied from one to three hours.

In each formal consultation, an introduction and background to the project were given with the aid of a slide presentation, then an early version of a public health classification, rendered through a Web browser, was demonstrated, concluding with

---

[22] Noy & McGuinness 2001.

[23] For more information see http://protege.stanford.edu.

[24] Noy & McGuinness 2001: 5-8.

the definition of public health. This was followed by a live collaborative session using the *Protégé* software, during which changes and additions to the class structure were made in real time. Lastly, participants were asked to identify further practical uses for a unified public health classification. An example of the agenda and other pre-consultation material that was sent to participants is in *Appendix B*.

The views, suggestions, and additional information captured in consultations were discussed by the Project Reference Group over a series of meetings and have informed the broad structure of the public health classification that is reported in *Section 3 Results*. The public health content experts who contributed are acknowledged in *Appendix B*.

An earlier version of this report was presented to, and discussed by, the National Public Health Information Working Group in March 2005, and this version reflects the feedback and directions given by that Group.

3 Results

This section presents the results of the process of scoping the domain to be covered by a public health classification. A number of boundary issues are discussed, areas of likely agreement identified, and potential practical applications for the classification are outlined. Version one of the public health classification is presented. Issues for further consideration are highlighted in boxes.

3.1 Scope and domain

3.1.1 Definition

The existing National Public Health Partnership definition of public health was adopted, as follows:

> Public health is the organised response by society to protect and promote health, and to prevent illness, injury and disability. The starting point for identifying public health issues, problems and priorities, and for designing and implementing interventions, is the population as a whole, or population sub-groups.[25]

Suggestions made during consultations that the Partnership definition should include references to '*evaluating*' and '*measuring or achieving outcomes'* were not adopted, as these were considered to be implicitly present in the definition.[26]

3.1.2 Boundary issues

Significant boundary issues were encountered in scoping the domain of public health, with disagreement among public health experts regarding where the boundaries are, or should be.

While most public health experts agreed, when pressed, that accounting for public health should include the activities of, and investments by, the non-health portfolios of governments (such as education and transport), local governments and non-government organisations (NGOs), current public health expenditure reporting is largely limited to that by State, Territory and Australian Government health portfolios.[27] One view was that the activities of other (non-health) sectors should only be counted when public health is their primary purpose (e.g. immunisation organised by local government). In practice there are major difficulties in capturing information on public health activities and expenditure by non-health sectors.[28]

The boundary between public health and clinical practice came up repeatedly in discussions about the scope of public health. In many situations preventive activities in clinical practice complement broader population-based activities. At what point do

---

25 NPHP 1998.

26 See *Appendix C* for additional information on this aspect of the Project.

27 With the exception of SA which has in the past included non-health expenditures by local government, etc, in public health expenditure reporting by AIHW.

28 A more fundamental difficulty is the time and expense to collect comparable information across all sectors.

they become part of 'the *organised* response by society to protect and promote health'? Organised interventions for promoting health and preventing illness, injury and disability include those aimed at whole populations that do not necessarily require any particular action on the part of individuals (**health protection** activities, e.g. the provision of clean water, clean air, sewage disposal), and those organised and delivered at the level of the population or sub-group, but requiring individuals to modify their behaviour (**health promotion** activities, e.g. the range of activities to reduce smoking in the community—regulations, media campaigns, organised quit lines etc). There was general agreement that health protection and health promotion are public health activities.

There was more debate in relation to **preventive services** delivered on a one-to-one basis to individuals. Such preventive services include screening, immunisation, and counselling and lifestyle advice to support healthy behaviour, as well as detection and management (through lifestyle changes or pharmacological means) of biological risk factors such as high blood pressure and high cholesterol. The perceived boundary between public health and clinical medicine is likely to change as new screening technologies and preventive medications become available.

Some public health practitioners argued that those individual preventive services related to **communicable diseases** (e.g. immunisation, contact tracing, treatment for STIs) form part of public health practice because they help to protect the health of the whole population, through herd immunity and reducing the spread of infection. A minority argued that immunisation is only a legitimate part of public health activity when it is delivered as part of a publicly organised program, such as through local government or school health services. The corollary of this point of view is that immunisations performed in general practice are not a public health activity. Alternatively, it was argued that childhood immunisation in general practice is simply the implementation strategy for an organised national approach to immunisation, one which is supported by special payments to GP's and the Australian Childhood Immunisation Register, with follow-up of those parents who do not comply.

With respect to **non-communicable diseases***,* early detection through screening is a preventive service delivered one-to-one to individuals. Some public health practitioners felt that this is only a public health activity when it is delivered through an organised program such as BreastScreen. Cervical screening is largely delivered through general practice, although, as with immunisation, the delivery of services in the private sector is underpinned by the National Cervical Screening Program (State and Territory recruitment programs, Pap smear registers, follow-up and reminder systems). The question is whether the taking of smears (in general practice) and the reading of smears (in laboratories), which are largely in the private sector, but essential to the implementation of the program, should be considered as public health activities.

On the other hand, opportunistic screening that is not part of an organised program, such as bone density screening for osteoporosis, was not generally considered to be a public health activity.

Even more contentious was the issue of the prevention and management of non-communicable diseases, through one-on-one counselling about lifestyle risk factors (e.g. smoking, poor nutrition, risky alcohol use and lack of physical activity), and the early detection and management of biological risk factors such as high blood pressure and high cholesterol in the prevention of heart disease and stroke. Many public health

practitioners regarded these activities as clinical practice. Some suggested that a distinction can be made on the basis of whether people have symptoms or signs of disease. For example, helping people to quit smoking would be considered a public health activity when they are symptom free, but part of clinical medicine if they have any symptoms or signs of disease or a history of previously diagnosed disease. Apart from any conceptual objections to such a distinction, it would be difficult to operationalise in practice.

Further along in the disease continuum, most public health practitioners classified the effective management of chronic disease, with the goal of minimising disability and reducing complications and hospitalisations, as belonging firmly in the zone of clinical medicine. For example, the prescribing of cholesterol-lowering medication by a general practitioner, even in an otherwise healthy person, would not be considered a public health activity (although a media campaign urging people above a certain age to have their cholesterol levels checked by their GP might be regarded as public health).

These boundary issues are set out for further consideration in *Box 2* in *Section 3.1.6*.

3.1.3 Potential uses

The Public Health Performance Project[29] envisaged that a unified classification for public health would be used to progress national public health expenditure reporting,[30] public health performance indicators,[31] and to build on the public health core functions developed by the National Public Health Partnership.[32]

Potential uses and practical applications for a public health classification, identified during phase one of the current project, are summarised in *Box 1*.

Box 1 Potential uses for a public health classification

- Explain what public health is
- Organise information to answer key public health questions
- Promote consistency in describing public health
- Improve data capture processes and the quality of reporting
- Contribute to higher-level classification and standards activities
- Structure and design information and communications
- Audit the spread of activity across the public health business cycle
- Build models of good public health practice
- Link research, policy and practice

---

[29] Owen & Jorm 2002: 8.

[30] AIHW 2004b.

[31] NHPC 2004.

[32] NPHP 1998.

A public health classification will help to **explain what public health** is in a way that is recognisable and understood by the average person. It will allow description of the functions of public health, issues of public health concern, the settings in which public health activities occur, the population groups targeted by public health interventions, the resources available to public health, and so on. The process of developing a classification has the potential to unite the sector and improve understanding of the breadth of the public health effort.

A public health classification can be used to organise information **to answer key questions for public health** that cannot be answered currently. While agreement on the scope of public health proved contentious during consultations, formulating questions that a competent public health classification should help to answer was somewhat easier. Questions like those shown in *Box 2* set a practical test for the classification.

Box 2 A public health classification should help answer questions like...

- How much was spent last year on the prevention of obesity?
- What is public health? What are the characteristics of public health?
- How is public health relevant to components of the human services delivery system?
- Why do public health unit costs differ across jurisdictions?
- Can we describe screening in clinical settings (e.g. Pap smears taken in GP surgeries)?
- What are the nature and cost of public health partnerships between health and other sectors?
- Can we replicate the output of other models (e.g. current public health expenditure reporting)?
- How much was spent on social marketing last year? [33]

As well as organising and integrating public health information, the development of a common classification will **promote consistency in describing public health,** through the standardisation of definitions and terminology. This will **improve data capture processes and the quality of reporting** (e.g. in expenditure and performance reporting). Promoting consistency will increase the ability to compare public health information over time and across jurisdictions.[34] There is potential for a public health

[33] Additional questions include other advocacy-type questions, such as: what is the relative expenditure on specific risk factors or diseases? What is the difference in expenditure on prevention of HIV/AIDS relative to other preventable diseases? Has health funding to preventive/promotive investments increased?. There are also boundary questions such as: can we describe the hospital interface with public health interventions (e.g. screening in hospitals)? Can we calculate expenditures in specific areas (e.g. product safety and protection, public health emergencies, education as a health promotive activity)? Competency questions can be used as a 'litmus test' to help determine whether the classification contains sufficient information to answer them, and whether the answers require a particular level of detail or representation of a particular area (Noy & McGuinness 2001: 5).

[34] Recent reporting of public health expenditures over several years has enabled such analyses for the first time (AIHW 2004b).

classification to be used to improve jurisdictional public health financial processes (e.g. budgeting, resource allocation) and accounting systems (e.g. through developing systems that can apportion public health activities to cost centres or to aggregate Treasury outputs).[35]

A public health classification will **contribute to higher-level classification and standards activities** through the potential membership of the Australian Family of Classifications. The development of the classification could 'fill out' the public health cells and embed public health more firmly into the 'health and related classifications matrix'.[36]

A public health classification can be used to **structure and design information and communications** (e.g. in designing websites, structuring resources, and planning report chapters). It has practical applications in building information systems, such as a database of public health projects, using the classification to create explicit, structured information to make meaning (as well as documents) accessible and shareable. One test application proposed was for a public health equivalent of the Semantic Web Environmental Directory (SWED).[37] This could be created through web-based, universally available tools, that make it easy for public health people to describe what they do, using the classes and terms from the public health classification. Other uses are based on a broad vision of a public health classification as signposting or semantically indexing a wide range of resources (including but not limited to: thesauri, dictionaries, terminologies and definitions, scientific papers, reports and other documents, legislation, policies, information databases and indexes, case studies, stories and vignettes).

A further use for a classification identified in consultations is to **audit the spread of public health activity, expenditure or investment**, across the business cycle – from health problem identification and assessment to program or intervention planning and design, through to implementation and evaluation of results. This suggestion arose out of concerns that public health activity is too heavily weighted towards implementation, and that there is insufficient evaluation of interventions, and learning from and progressing beyond pilots. A related use is to examine the spread of all public health investments, for example, by Australian, state, territory, and local governments, NGOs and other investors; the links to employment and education; and public health investment by, and outcomes in, other sectors such as transport and housing.

---

[35] For instance, a current difficulty is multiple attribution of multi-function programs – the so-called '220% problem'. We envisage rules that apportion public health programs across functions or issues, much as burden of disease studies calculate attributable risk factors.

[36] The Classifications and Terminologies Working Group of the National Health Information Group has scoped the concept of a 'family of health and related classifications' as an applications matrix drawing on available standard classifications, both Australian and international (CTWG 2004: 9-10). The World Health Organization has published a similar matrix as 'A Schematic representation of the World Health Organization Family of International Classifications' (WHO 2004: 8-9).

[37] SWED can be visited at http://www.swed.org.uk/swed/index.html. Sir Tim Berners-Lee, who is often referred to as 'the father of the World Wide Web', describes SWED as 'a prototype of a new kind of directory of environmental organisations and projects... Rather than centralising the storage, management, and ownership of the information, SWED simply harvests data and uses it to create the directory' (Frauenfelder 2004).

A classification can potentially be used to help **build models of good public health practice** that describe the program logic for public health activities, including specification of the links between activities, expenditure and outcomes. Another suggested use for a classification is in developing a continuous improvement model to ensure that public health learns from what it does.[38]

Lastly, the classification was considered to have the potential to **link public health research, policy and practice**, by facilitating use of a common language, and the linkage of information across these domains.

3.1.4 Who will use and maintain the classification?

Potential users of a public health classification, who were identified along with the practical applications discussed above, are the various levels of government and other sectors that have an investment in public health. Other users include academics and students, researchers, evaluators, those involved in policy formulation, and anyone with an interest in public health.

The Australian Institute of Health and Welfare has indicated an interest in the longer-term development and maintenance of a public health classification.

3.1.5 Principles of development

During the development of the public health classification, the following principles of development were determined and agreed:

- The classification system should be multi-dimensional to be able to represent the multi-dimensional nature of public health.
- Different dimensions are of equal importance to public health and a range of the most important need to be considered and developed concurrently.
- Existing classification systems of relevance (including Australian and international standards) should be used wherever possible in the multi-dimensional structure of the classification system.
- The system should be inclusive (rather than exclusive) and deliberately broad at the top levels. Boundaries can be set (or moved) as needed for particular practical applications; they should not be used to restrict or hinder the development of a broad and inclusively scoped classification.

3.1.6 Issues for further consideration

In addition to the definitional issues raised and discussed in *Section 3.1.2* above, public health experts consulted in phase one of the project raised the important issue of whether the name of the project domain should be 'population health' or 'public

[38] An example is the Shewhart or Deming Plan-Do-Check-Act cycle. Deming (1993) recommended that business processes be placed in a continuous improvement loop so that managers can identify and change the parts of the process that need improvements. Many public health processes are based on continuous improvement variants, for example, the public health 'surveillance loop'.

health'.[39] How is the domain that public health currently works in, best described? Is 'public health' subsumed in 'population health'? Or is a 'population health approach' merely one aspect of public health practice today?

The concept of population health has its origins in the Canadian Lalonde Report in 1974, which promoted the (then radical) idea that health and well-being involve more than the health care system, and that the adoption of healthier lifestyles, and improvements in people's social and physical environments, would be the principal means of improving the health of Canadians in the future.[40] Population health, as a way of acting on the social and economic forces that structure health, builds on a tradition of public health and health promotion that goes beyond a focus on the medical, biological or lifestyle problems of individuals.[41]

The decision on what to call public health is partly semantic, as the domain called 'public health' has changed over time. 'Classical' or 'traditional' public health had an external, environmental focus and produced major infrastructure projects such as sewage and safe drinking water systems, and other improvements to the human environment. *Figure 3* shows changes in the conceptualisation of public health over time in two axes: *population–individual* and *proactive–responsive*. In the figure, quadrant D describes the 'new' public health (and 'social' health, with a health equity focus) as a proactive population approach.

Figure 3 Conceptions of public health over time (B-D)

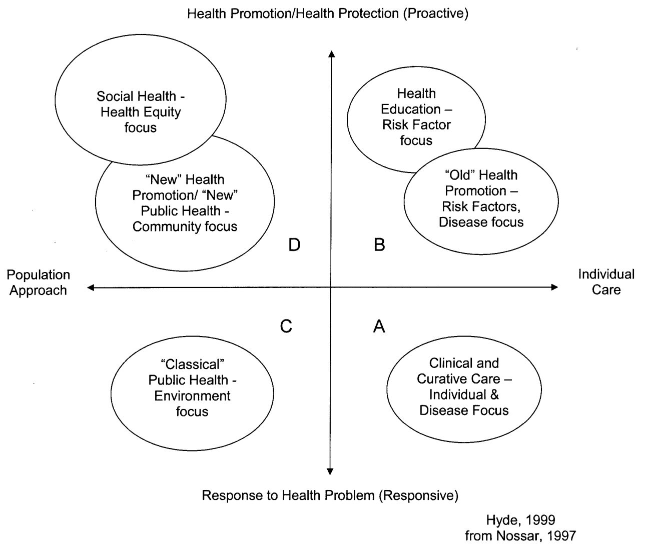

A population health approach can be defined as a subset of public health with a whole-of-population focus,[42] or as containing both public health and other health services.[43] Population health is not the only term that is sometimes misleadingly

[39] An alternative would be to include both terms in the domain name.

[40] Lalonde 1974.

[41] Hayes & Dunn 1998. The population health approach is not without its critics, some of whom argue that it has been captured by the focus on the problems of individuals (e.g. overweight persons), while losing sight of the larger issues (e.g. obesogenic environments) (Raphael & Bryant 2002).

[42] Bennett 2003: 12.

[43] For instance, a 'population health approach describes a comprehensive health system which ranges from public health at one end to individual health care at the other' (Buckett & Hunter 2004). Fraser (2005) conceptualises population health as 'the health of a defined population, or a field of study that links health outcomes, determinants of health, and interventions' but notes that it is an 'ill-defined term' in the literature. The term public health has competing definitions, but is considered by many health professionals to be 'broader and more encompassing than population health' (Fraser 2005: 177).

contrasted with public health and adds to the confusion about what public health is. *Figure 4* shows how such confusion can arise from the *intersection* of public health with other perspectives on health—such as, a population health approach, and definitions of preventive health, and primary health care.

Does it matter what the domain is called? The term 'population health' was preferred over 'public health' in several consultations during phase one of the project. A sampling of jurisdictional health departments showed that population health has overtaken public health in popularity as the name for the relevant organisational units (see *Appendix E*).

Figure 4 Overlapping concepts relating to public health

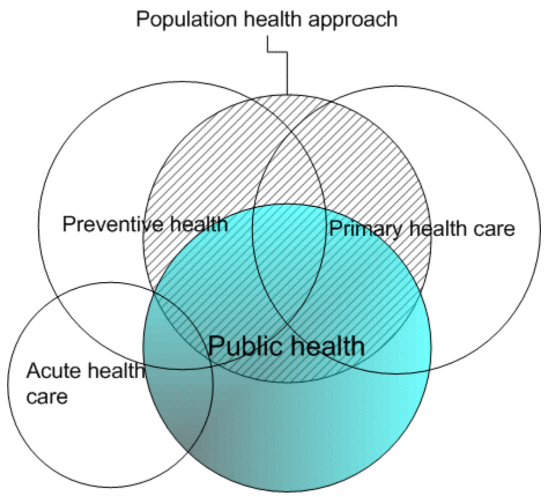

At other consultations, the term public health was strongly preferred to population health as the name of the domain (although a 'population health approach' was allowed as a method *used* by public health). There is also a widespread view among public health experts that the general public commonly confuses or equates public health with public hospitals, or the health system funded from the public purse. Some practitioners saw the rise in the popularity of the term 'population health' as an opportunity to gain agreement on an all-encompassing definition and to replace the often misunderstood term 'public health'.

As was noted in the discussion in *Section 3.1.2* above, the boundary between public health and clinical medicine is contentious, and both the boundary and the components included in each are likely to change over time. There may never be complete agreement by all experts, but the act of making components and boundaries explicit can at least facilitate discussion on these difficult issues that are summarised as discussion points for further consideration in *Box 2*.

Box 2 Issues of definition for further consideration

**Is the definition of public health agreed?**	'Public health is the organised response by society to protect and promote health, and to prevent illness, injury and disability. The starting point for identifying public health issues, problems and priorities, and for designing and implementing interventions, is the population as a whole, or population sub-groups.' (NPHP 1998)
**Issues**	▪ What is the preferred name for the domain of public health today (population health, public health, public and population health)?<br>▪ How is 'organised response' defined? Is there agreement on the following examples of organised response?<br>a. The breast cancer screening programme supervised by BreastScreen Australia;<br>b. Screening for cervical cancer by GPs underpinned by registers, recall systems, and target population monitoring;<br>c. GPs undertaking opportunistic screening for high cholesterol, in accordance with published National Heart Foundation guidelines, in patients consulting them for an unrelated matter?<br>▪ How is public health differentiated from clinical treatment services? When are treatment services - for example, treatment of sexually transmitted diseases or tuberculosis - part of public health?<br>▪ Does the place of delivery of services determine that a service is or is not a public health service? For example, is an immunisation delivered in a dedicated local government or school immunisation clinic different from an immunisation delivered in a hospital emergency department?<br>▪ Should the domain of public health be solely within health or should it include specific activities of other sectors (e.g. education, transport, local government) that have public health as a primary purpose? Or as a secondary purpose?

A checklist approach

One suggested response to these questions is for a checklist approach that operationalises the agreements realised in scoping the public health domain. This could be used to determine whether an activity is public health or clinical care, for instance. The checklist components could be weighted, so that an activity that meets one 'must-have' and two out of three other criteria is defined as public health.

The checklist could test whether the activity is preventive, (e.g. primary or secondary reason for service is to prevent the need for acute care; treatment for sexually transmitted disease is to prevent transmission of disease); whether it benefits a population (this does not preclude services to individuals - the benefit could be to an individual *and* a population, e.g. immunisation); whether a public health response is required *in addition to* (any) individual treatment response required (e.g. assess area for contaminant after individual exposure, check cooling towers in response to case of Legionnaires disease, trace contacts of person diagnosed with infectious disease); whether it is an organised response, for instance, in response to a disaster, over time (e.g. immunisation register), or in scale (e.g. screening across the nation, quality assurance through pathology reference laboratories).

3.2 Version one of a public health classification

3.2.1 Top-level classes and working definitions

The most important dimensions (or top-level classes) revealed in an analysis of the National Public Health Partnership *public health core functions*[44] were the functions of public health,[45] and the methods that public health uses to achieve those functions.

A selection of other candidate top-level classes was made in order to focus the project. Those initially chosen for detailed examination were:

- public health functions and activities or programs that funds buy (e.g. public health expenditure activities);
- determinants of health, health risk and protective factors (e.g. socio-economic determinants, behavioural factors);
- disease, disability, and injury areas (e.g. vaccine preventable diseases) that determine intervention targets; and
- the public health 'toolkit'—methods, tools, and bodies of knowledge, both those specific to public health (e.g. epidemiology, health promotion techniques) and those used by but not specific to public health (e.g. management methods, policy development frameworks).

These potential classes underwent extensive development and revision and are shown in *Figure 5* as they stand at the conclusion of phase one of the project (working definitions are in *Table 2*). Potential classes that were identified but not selected for detailed examination are discussed in *Section 3.2.5.*

Public health practitioners expressed both broad and narrow views of what a classification system for public health should include. These views reflect the range of practical applications they identified (detailed in *Section 3.1.3)*, and their underlying requirements. For instance, for health expenditure reporting, mutually exclusive activity categories at meaningful expenditure levels are required. From a health promotion viewpoint, the ability to model the public health business cycle, and to identify gross expenditure proportions for different elements (e.g. design, implementation, evaluation) are equally important.

There was however, consensus among the public health experts consulted, that a public health classification should be **multi-dimensional**, and there was broad agreement on the top-level classes that should be included.

There was agreement that public health '**functions**' form an important class, although there was some confusion regarding whether functions refer to the *purposes* of public health activities or the *methods* of intervention by which public health achieves its aims (see working definitions in *Table 2*).

---

[44] NPHP 1998.

[45] The word 'function' is used here in the sense of 'the purpose, role or use of something'; thus, the *function* of public health is 'to protect and promote health, and to prevent illness, injury and disability' (NPHP 1998).

There was also wide agreement that both ‘**health issues**’ and ‘**determinants of health**’ are central to public health, although there are differing views on the relative importance of individual determinants and how they should be structured at lower levels of the classification. The inclusion of a ‘**settings**’ class was also generally agreed.

Figure 5 Classification of public health: top-level classes

FUNCTIONS
HEALTH ISSUES
DETERMINANTS OF HEALTH
METHODS
SETTINGS
RESOURCES & INFRASTRUCTURE

The project involved extensive discussion and work regarding how to define the practice of public health, the methods and strategies used in public health interventions, and the bodies of knowledge that these draw on. There were two strong perspectives on what should be included in a classification. One perspective was restrictive and would narrow the scope of a ‘**methods**’ class to those methods that are *peculiar* to - or only used by - public health (e.g. population-based epidemiology, health promotion, environmental risk assessment). The other focus was on capturing *all methods* used by public health, including those that, while not specific to it, are employed by public health workers in the normal course of their work (e.g. administration, management, policy development).

A ‘**resources**’ class was elevated in importance when consultations reinforced the importance of the many types of infrastructure on which today’s public health relies: physical infrastructure (e.g. sewers, public health laboratories), organisational infrastructure (e.g. partnerships, legislative and regulatory systems), logistical infrastructure (e.g. vaccine cold chains) - systems that are seen by some as ‘joined up’ resources. There were diametrically opposed views of whether infrastructure was a subclass of resources or vice versa. In the short term this has been dealt with by amalgamating the two into a ‘**resources *and* infrastructure**’ class.

In consultations many public health experts wanted to add a ‘**policy**’ class. There are several elements to be described. One element is the public health work of *developing* healthy public policy. Whether or not policy is implemented, substantial work goes into its development, and its availability can provide a head start for action on a health issue that becomes of interest. Information on existing public policy that has an impact on public health is considered by some as important to collect and integrate—especially in the absence of a national public health policy. Some public health experts were comfortable with the concept of ‘policy’ as a resource or as part of the public health infrastructure, however others were strongly negative – they saw that as putting policy too low in the class hierarchy. This reveals the tendency to see the top-level classes listed in *Figure 5* as a hierarchy of the factors of most importance to public health, in which case, where is policy? Where are population groups? The discussion in *Section 3.2.3,* which is illustrated in *Figure 7,* addresses these questions.

Similarly, the addition of an ‘**outcomes**’ class was identified as important at almost every consultation, reflecting a view that outcomes (i.e. outcome indicators and their reporting[46]) are necessary to ‘close the loop’ and complete the program logic for

[46] For example, public health system performance measures and public health expenditure reporting.

public health. This reflects a tendency to see the top-level classes listed in *Figure 5* as a program logic or cycle (rather than a hierarchy of important factors) that requires information on outcomes to complete the cycle. An alternative view on the treatment of outcomes in a public health classification is that they are already captured in the classes of 'health issues' and 'determinants of health'. *Section 3.2.3* also addresses these issues.

Table 2 Classification of public health: top-level classes and working definitions

Top-level classes	Working definitions
Functions	Public health functions. The purpose of public health interventions, actions, activities and programs.
Health issues	Health, and well-being issues that affect health ('issues' includes: concerns, topics, problems). Health is defined (by the WHO) as 'a state of complete physical, mental and social well-being and not merely the absence of disease or infirmity'.
Determinants of health	Factors that influence health status and determine health differentials or health inequalities. They include, for example, natural, biological factors, such as age, sex and ethnicity; behaviour and lifestyles, such as smoking, alcohol consumption, diet and physical activity; the physical and social environment, including housing quality, the workplace and the wider urban and rural environment; and access to health care.[47]
Methods	The methods used by organised public health interventions (actions, activities, programs, services) to protect and promote health and prevent illness, injury and disability, that are designed to change population exposure, behavioural or health status.
Settings	Settings in which public health activities and interventions take place, institutional and social environments, partnerships, and locations (e.g. schools, local government, hospitals, workplaces).
Resources and infrastructure	Resources and infrastructure, 'the means available for the operation of health systems, including human resources, facilities, equipment and supplies, financial funds and knowledge'.[48] It includes both person-time and calendar time.

3.2.2 Public health functions

Considerable development of the functions[49] or purposes of public health took place during phase one of the project. As discussed above, the National Public Health Partnership (NPHP) *public health core functions*[50] were analysed and distilled into the individual functions of the 'functions' class of version one of a public health

[47] Based on WHO 2005, citing Lalonde 1974; Labonté 1993.

[48] WHO 1998a.

[49] Function is defined as 'the purpose, role or use of something'; thus, the *function* of public health is 'to protect and promote health, and to prevent illness, injury and disability' (NPHP 1998).

[50] NPHP 1998.

classification presented in this report (the proposed treatment of other components of the *public health core functions* is shown in *Table 5*).[51] The functions as scoped at the end of phase one of the project are shown in *Table 3* and their working definitions are given in *Table 4*.

Table 3 Classification of public health: overview of the functions class

Top-level class	Level 2 subclasses	Level 3 subclasses
**Functions:** **Primary functions (ends)**	Assess health of populations	Monitor health<br>Evaluate health risks and benefits<br>Assess health inequalities
	Protect from threats to health	Prepare for threats to health<br>Respond to threats to health<br>Control and mitigate risks to health
	Promote health and prevent disease, disability and injury	Promote health and wellbeing<br>Prevent the occurrence of disease, disability and injury<br>Detect disease, disability or injury in its early stages
**Instrumental functions (means to achieve ends)**	Ensure public health capability	Develop and maintain the public health workforce<br>Develop and maintain public health infrastructure<br>Build public health partnerships
	Build the evidence base for public health	Conduct public health research<br>Evaluate public health interventions

Both primary and instrumental functions are of importance in conceptualising public health. Primary functions are *ends* in themselves, while instrumental functions are *means* to those ends, as without primary functions there would be no need to 'ensure public health capability', for instance. Instrumental functions were also described in consultations as supporting, underpinning, or crosscutting functions, as all primary functions rely on them and they do not belong exclusively to any one of the primary functions.

Although the instrumental 'build the evidence base...' function could be included in 'ensure public health capability', it is shown separately because building an evidence base and moving towards decisions informed by evidence are key features of the current context for public health.

Other functional classifications of public health were explored during the course of the project, including that portion of the OECD *System of Health Accounts* that is relevant to public health.[52] The OECD classification has a similar mix of classes within functions as do the *public health core functions*, but excludes environmental

---

[51] The public health core functions (NPHP 1998) are shown in *Table 5*.

[52] 'Prevention and public health services' defined (in part) as services 'mainly of a preventive nature and ... publicly provided' which include 'special public health services such as blood-bank operation, public health service laboratories, and population planning services' (OECD 2000: 44).

health, and was structurally not helpful.[53] A functional division that followed the distinctions between primary, secondary and tertiary prevention was also explored but the classification was confusing and difficult to apply, and there are arguments that tertiary prevention in particular, has more relevance to clinical treatment services than to public health.

A comparison of the public health functions of selected other nations (see *Figure 6*) shows that, in the UK for instance, both primary (e.g. health promotion and disease prevention programs) and instrumental (e.g. development and maintenance of a public health workforce) functions are prominent, while the public health functions of Canada and the Americas are limited to primary functions.[54] Both the UK core functions[55] and the USA essential public health services include a specific (instrumental) partnership function for public health. In Australia, the essential importance and defining nature of inter-departmental, inter-governmental, inter-sectoral and other partnerships, in the work of public health was made clear in the expert consultations. Accordingly, version one of a public health classification proposes 'build public health partnerships' as a subclass of the 'ensure public health capability' instrumental function (see *Table 3*).

Figure 6 Other national public health functions

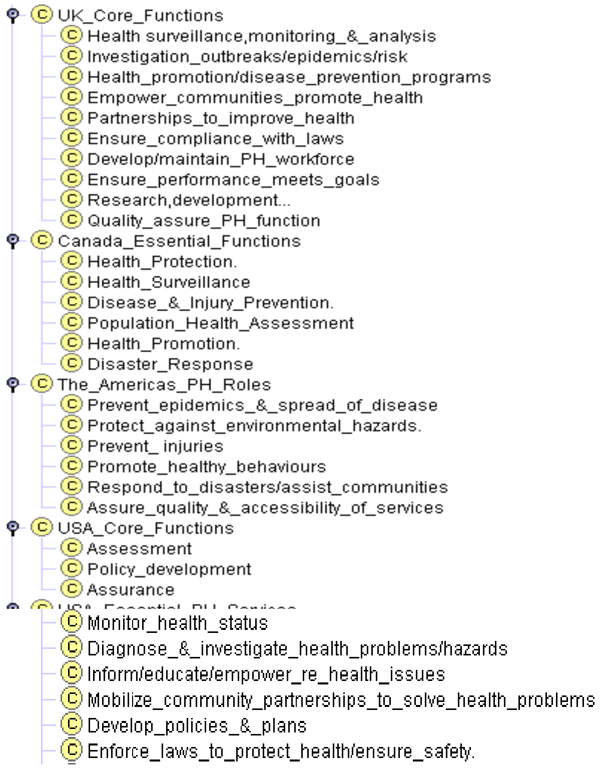

Sources: CMO UK 2003, National Advisory Committee on SARS & Public Health 2003, PAHO 2002, IOM 1988, US DHHS 1995. The picture is from a *Protégé* version of the top levels of existing classifications of interest.

---

[53] Dimensions used by the OECD are: population groups, service types, disease types, and settings.

[54] A recent review conducted by WHO (2003), describes comparable 'essential public health functions' as 'a set of fundamental activities that address the determinants of health, protect a population's health, and treat disease... public health functions represent public goods, and... governments would need to ensure the provision of these essential functions, but would not necessarily have to implement and finance them. They prevent and manage the major contributors to the burden of disease by using effective technical, legislative, administrative, and behaviour-modifying interventions or deterrents, and thereby provide an approach for *intersectoral action for health* [that] *stresses the importance of numerous different public health partners*. Moreover, the need for flexible, competent state institutions to oversee these cost-effective initiatives suggests that the institutional capacity of states must be reinforced' (Yach 1996 cited in WHO 2003: 1, our *italics*).

[55] The full description of this function is 'Creating and sustaining cross-Government and intersectoral partnerships to improve health and reduce inequalities' (CMO UK 2003; see chapter 3).

The UK core functions also include a specific (instrumental) research function. In Australia, although 'public health research' is one of the nine core public health activities for which public health expenditure is reported,[56] there is no corresponding function in the NPHP *public health core functions*. Version one of a public health classification proposes 'conduct public health research' as a subclass of the 'build the evidence base for public health' instrumental function (see *Table 3*).

A health surveillance function is common to both the UK and Canada. It is broadly specified in the UK as 'health surveillance, monitoring and analysis', while in Canada the function of 'population health assessment' is specified separately, in addition to 'health surveillance'.[57] In Australia, the first of the nine NPHP *public health core functions* is 'assess, analyse and communicate population health needs...' (and is proposed as 'assess health of populations' in version one of a public health classification - see *Table 3*), although expenditure on this public health activity is not currently reported in an identifiable manner.

A quality assurance function is specific for public health in both the USA and the UK. Whether such a function is pertinent to public health in Australia is a matter for discussion and has not been canvassed in consultations.

Working definitions of functions – a work-in-progress

Working definitions of the public health functions proposed in *Table 3* are given in *Table 4*. The working definitions are based on NPHP *public health core functions*[58] and extensive discussion during the project. Some of the major strands that emerged in discussions, and their impact on the working definitions, are reported below.

The 'promote health and prevent disease, disability, and injury' function was initially cast as two functions, with 'promote better health' separate. In examining the mission statements and goals of health promotion and prevention units across the jurisdictions it was clear that there was no hard boundary between the promotion of health and the prevention of disease, disability and injury. In consultations it was suggested that the two functions should be married together as the distinction is increasingly blurry in practice. They have thus been joined as one function at the top level.

'Develop healthy public policy' was initially classed as a subclass of a 'promote better health' function. In consultations it was pointed out that this function, method or strategy was cross-cutting, applying to all primary functions, and should not be singled out as belonging only to one function, or as separate to all other functions. 'Policy development' was thus classed as a 'method' of intervention so that it can be applied to any or all of the public health functions in an additional dimension.

---

[56] AIHW 2004b.

[57] The post-SARS Canadian view is that: 'Among the *functions of public health* are health protection (e.g. food and water safety, basic sanitation), disease and injury prevention (including vaccinations and outbreak management), population health assessment; disease and risk factor surveillance; and health promotion. The public health system tends to operate in the background unless there is an unexpected outbreak of disease such as SARS or failure of health protection as occurred with water contamination... An effective public health system is *essential to preserve and enhance the health status of Canadians, to reduce health disparities, and to reduce the costs of curative health services.* [It] also plays a key role in disaster and emergency response' (National Advisory Committee on SARS & Public Health 2003).

[58] NPHP 1998.

Table 4 Classification of public health: working definitions of functions subclasses

Functions subclasses	Working definitions
Assess health of populations	Monitor and analyse health and determinants of health in populations, assess the impacts of policies, interventions, and environmental exposures.
Monitor health	Monitor and analyse levels of health and its determinants in populations to identify and predict trends and emerging issues ('Assess health inequalities' would be a further subclass of this).
Evaluate health risks and benefits	Evaluate adverse and beneficial effects related to health and social policies and interventions, and environmental exposures.
Assess health inequalities	Assess inequalities in health (level and distribution) and health gain to target interventions to improve the health of the worst-off sub-populations.
Protect from threats to health	Protect from, and prevent, external threats to public health.
Prepare for threats to health	Identify and prepare for potential threats to health (including communicable diseases, environmental hazards, bio-terrorism and new patterns of exposures e.g. arising from ecological change).
Respond to threats to health	Respond to threats to health (including communicable diseases, environmental hazards, bio-terrorism and other disasters).
Control and mitigate risks to health	Minimise or reduce the severity of risks to health (includes setting and monitoring of standards for e.g. food, air and water quality and other potential hazards, also harm minimisation measures).
Promote health and prevent disease, disability and injury	Promote health and wellbeing, prevent the occurrence of disease, disability and injury; and detect disease in its early stages, through organised efforts that target populations.
Promote health and wellbeing	Promote better health and well-being as it affects health (e.g. community development and community empowerment initiatives clearly differentiated from 'Prevent the occurrence of...').
Prevent the occurrence of disease, disability and injury	Prevent the initial occurrence of disease, disability and injury (e.g. population-level campaigns to promote physical activity, tobacco control, seat belt legislation).
Detect disease, disability or injury early	Detect disease, disability and risk of injury early and initiate prompt management or response (e.g. screening for cancers, newborn hearing screening).
Ensure public health capability	Ensure adequate public health capacity and responsiveness by maintaining and developing the public health workforce and infrastructure, and building partnerships with other sectors of society.
Develop and maintain the public health workforce	Train, maintain and develop the public health workforce.
Develop and maintain public health infrastructure	Build, maintain and develop public health infrastructure, including physical, organisational, legislative, communication and informational, logistical, and other systems forming the public health infrastructure.
Build public health partnerships	Build and maintain public health partnerships with other sectors and the community, to work together on shared issues and undertake actions towards agreed targets.
Build the evidence base for public health	Increase and enhance the bodies of knowledge and evidence that inform public health practice (e.g. research, research synthesis, evaluation).
Conduct public health research	Conduct public health research.
Evaluate public health interventions	Evaluate public health interventions.

There was also a view that a 'promote better health' function should be expanded to 'enhance health and quality of life,' to incorporate the concepts of: (1) effort from non-health sectors that affects public health, and (2) quality of life and health maintenance (rather than improvement) where the presence of disease makes health improvement an inappropriate aim. Agreement on these definitional extensions was lacking in further consultations and they have not been adopted.

The working definitions in *Table 4* are shown as they stand at the conclusion of phase one of the project. They should be regarded as a work-in-progress and a point to move forward from, rather than the definitive last word on the public health functions.

Correlation with the NPHP public health core functions

The relationship between the 'functions' class and other top-level classes in version one of a public health classification and the National Public Health Partnership (NPHP) *public health core functions*[59] is shown in *Table 5*. The table illustrates how the public health classification can be used to achieve a functional equivalence to the several dimensions implicit in the NPHP *public health core functions*.

The multi-dimensional *core functions* can be classified using different top-level classes of the classification (e.g. 'health issues', 'methods'), and instances (see *Figure 7)*. For example, the **function** or purpose of core function two (shaded in *Table 5*) is to '*Prevent and control communicable and non-communicable diseases and injuries'* using the public health intervention **methods** of '*risk factor reduction, education, screening, immunisation and other interventions*'.

Table 5 Functional equivalents of the public health core functions

	**Public health classification functional equivalent (b)**	
Public health core functions (a)	**'Functions' subclass/es**	**Additional classification (c)**
1. Assess, analyse and communicate population health needs and community expectations.	Assess health of populations	
2. Prevent and control communicable and non-communicable diseases and injuries through risk factor reduction, education, screening, immunisation and other interventions.	Protect from threats to health<br>Promote health and prevent disease	*Risk factor reduction, education, etc* classified as 'methods', and instances described as Interventions. *Communicable and non-communicable diseases etc* classified as 'health issues'.
3. Promote and support healthy life-styles and behaviours through action with individuals, families, communities and wider society.	Promote health and prevent disease<br>Ensure public health capability: Build partnerships	*Action with individuals, families, communities etc* classified as 'methods', instances described as Interventions, and *families, communities* described as Population Group instances.
4. Promote, develop and support healthy public policy, including legislation, regulation and fiscal measures.	All.	*Public policy* measures classified as 'methods', and instances described as Interventions.

(a) NPHP 1998. (b) This project, see *Tables 3* and *4,* this report. (c) Classification by other classes of the multi-dimensional public health classification (e.g. health issues, determinants of health, methods) or as instances of things to be classified (e.g. population groups, interventions).

[59] NPHP 1998.

Table 5 Functional equivalents of the public health core functions, continued

Public health core functions (a)	Public health classification functional equivalent (b)<br>'Functions' subclass/es	Additional classification (c)
5. Plan, fund, manage and evaluate health gain and capacity building programmes designed to achieve measurable improvements in health status, and to strengthen skills, competencies, systems and infrastructure.	Ensure public health capability<br>Build the evidence base for public health	*Plan, fund, manage and evaluate* classified as 'methods', and instances described as Interventions. *Programmes* described as instances of Public Health Activities.
6. Strengthen communities and build social capital through consultation, participation and empowerment.	Promote health and prevent disease<br>Ensure public health capability	*Consultation, participation and empowerment* classified as 'methods', and instances described as Interventions.
7. Promote, develop, support and initiate actions which ensure safe and healthy environments	Protect from threats to health<br>Promote health and prevent disease	*Actions* described as instances of Public Health Activities.
8. Promote, develop and support healthy growth and development throughout all life stages	Promote health and prevent disease	*Healthy growth and development* classified as a 'health issue' (e.g. 'health and well-being'). *Life stages* described in Population Groups.
9. Promote, develop and support actions to improve the health status of Aboriginal and Torres Strait Islander people and other vulnerable groups.	Assess health of populations: Assess health inequalities<br>Protect from threats to health<br>Promote health and prevent disease	Individual *vulnerable groups* described as Population Groups classified by other classes (e.g. person-level demographic descriptors in 'determinants of health'). *Actions* described as instances of Public Health Activities.

(a) NPHP 1998. (b) This project, see *Tables 3* and *4,* this report. (c) Classification by other classes of the multi-dimensional public health classification (e.g. health issues, determinants of health, methods) or as instances of things to be classified (e.g. population groups, interventions).

Core function nine (shaded in *Table 5*) *'Promote, develop and support actions to improve the health status of Aboriginal and Torres Strait Islander people and other vulnerable groups'* identifies important target **populations**, rather than describing a separate function of public health. Functions, methods, and population groups thus form three distinct dimensions (among many) of interest in a multi-dimensional classification of public health.

Few of the nine public health core functions have a one-to-one relationship with the functions of the public health classification, if the functional equivalence shown in *Table 5* is accepted.

Core function four *'Promote, develop and support healthy public policy, including legislation, regulation and fiscal measures'* requires special mention, as it is shown as relevant to all the functions of the public health classification. It is proposed that public policy measures are methods to address *all* functions rather than a function in their own right. 'Public policy development' is thus shown as a separate method in *Table 6*, as is 'legislation and regulation' (which some see as enacted policy). Public health activity using these methods can have a major impact on population health. Examples include the impact on population smoking rates of legislation, regulations, and fiscal measures implemented under the policy umbrella of the Tobacco Control Strategy.

Although the multi-dimensional structure of the public health classification is quite different to the flat list structure of the *public health core functions*, its classes can be used in a functionally equivalent way to classify and describe the functions and other important dimensions of public health.

3.2.3 Other top-level classes

The public health dimensions currently scoped, and their top-level classes are shown in *Table 6*. The 'functions' class has been discussed in *Section 3.2.2*. While there was reasonable agreement among the public health experts consulted over the top levels of the classes of 'health issues' (although its name was debated), 'determinants of health', and 'settings', the remaining classes are in the early stages of development and have not yet been subject to detailed consideration. The 'methods' class, in particular, established to describe the methods of public health intervention, is at an early stage of development.

Table 6 Classification of public health: top levels of all classes

Top-level class	Level 2 subclasses		
Functions<br>*Primary:*	Assess health of populations<br>Protect from threats to health	Promote health and prevent disease, disability and injury	
Instrumental:	Ensure public health capability	Build the evidence base for public health	
Health issues	Health and well-being<br>Diseases and conditions	Injury<br>Disability and functioning	
Determinants of health	Environmental<br>Person-level	Socioeconomic<br>External causes of injury	Health system
Methods	Advocacy & lobbying<br>Communicable disease control specific<br>Community action<br>Community development<br>Counselling<br>Diagnosis<br>Directed investment<br>Environmental monitoring<br>Epidemiologic methods<br>Exercise of capabilities<br>Food safety methods<br>Health education<br>Health impact assessment<br>Immunisation<br>Infection control	Legislation & regulation<br>Lifestyle advice<br>Management of biological risk<br>Monitoring and surveillance<br>Personal skills development<br>Political action<br>Public policy development<br>Radiation safety methods<br>Remediation of environment methods<br>Research & evaluation<br>Road safety methods	Screening to detect disease/risk factors<br>Social action<br>Social marketing<br>Training and workforce development methods<br>Treatment methods<br>Urban planning methods<br>Vector control methods<br>Waste management methods<br>Other methods of intervention
Settings	Educational settings<br>Healthcare settings<br>Local government and communities settings	Home settings<br>Workplace settings<br>Transport settings<br>Other settings	Includes LOCATIONS – classification of geographical areas (e.g. postcodes).
Resources and infrastructure	Administrative infrastructure<br>Funds<br>Information systems<br>Legislative infrastructure<br>Organisational systems	Partnerships<br>Physical infrastructure<br>Policies<br>Technical infrastructure	Time<br>Workforce<br>Workforce development capacity

While population groups are important, it was generally agreed that they are not a top-level class in a public health classification. As the targets of public health interventions, instances of population groups can be described by other classes in the

classification, such as the person-level demographic descriptors in the 'determinants of health' class (e.g. age, sex). There was also agreement that stakeholders and partners, although important in the work of public health, did not warrant their own top-level class. As with population groups, they may also be described by other classes in the classification. This distinction is illustrated in *Figure 7*, which distinguishes between classes in the classification (circles) and items to be classified (heptagons). The latter include (but are not restricted to) **public health activities** and programs (centre), public health **interventions**, **public policies**, **outcomes** (indicators that are useful for public health purposes, and those that are nationally reported), **population groups**, **partners and stakeholders** in the public health effort.

Figure 7 also shows whether suitable classifications exist for use by the top-level classes, or whether they need to be developed. Existing classifications (e.g. Australian standards, international classifications of diseases, functioning and disability, external causes of injury) are available to classify major parts of the 'health issues', 'settings' and 'resources' classes. The National Public Health Information Working Group has determined that further development of classifications for the 'functions', 'determinants of health' and 'methods' classes are a required priority for the second phase of the project.

Figure 7 A model of public health classification

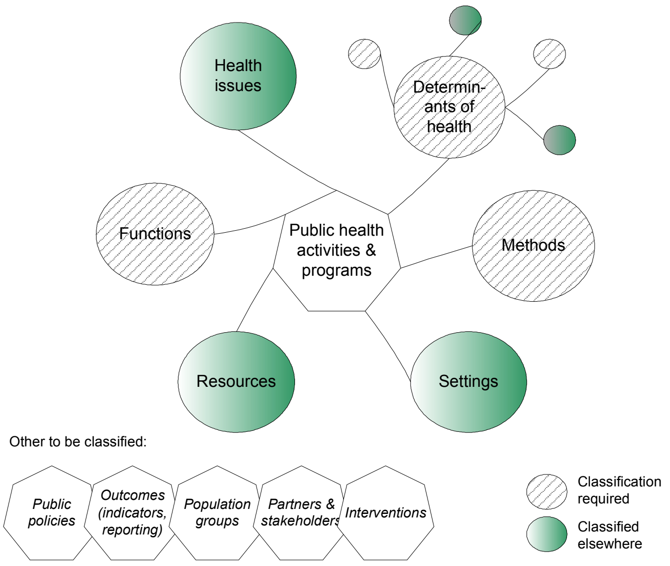

Not all public health experts will agree with the constituent parts of the classes as they stand, and some important parts are undoubtedly missing. The project anticipates feedback on these issues through making these results more widely available.

3.2.4 Using the public health classification

During phase one of the project, some of the practical uses that had been identified were developed in a small way in order to test the usefulness of the classification. Two examples—of public health activity from national public health expenditure reporting, and details of public policies—are detailed below. Information on some recent developments of interest in public health classification in the UK can be found at the end of *Appendix B*.

Example 1 Public health activity

A selection of public health activities from public health expenditure reporting were classified using the top-level classes of the public health classification. The detail of an example public health activity is shown in *Figure 8*. The (C) symbol denotes classes and subclasses, while the (I) symbol denotes 'instances' or individual cases,

for example, an individual public health activity, partner, stakeholder, or population group.

Figure 8 Detail of a classified public health activity

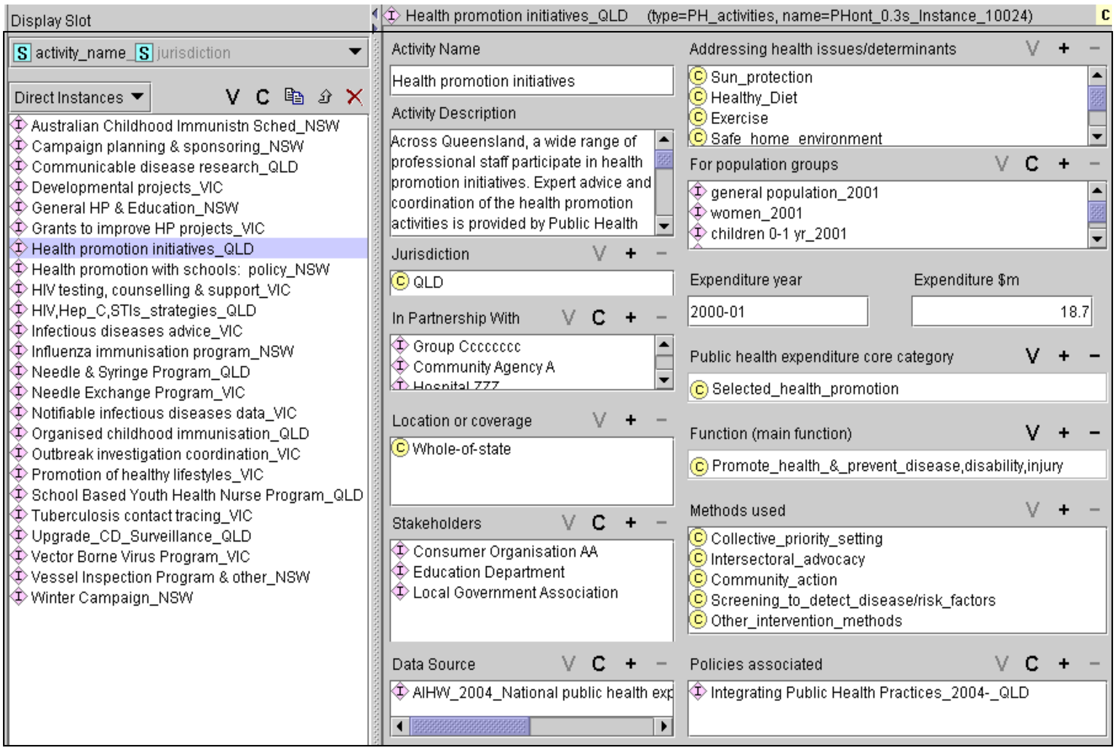

On the left of the figure is a list of public health activities extracted from the latest public health expenditure report[60], and to the right are the details of a selected activity, characterised by a number of 'slots' or attributes of the activity. The selected activity is Queensland's 2000-01 health promotion initiatives, on which $18.7 million was expended. The example shows the variety of *health issues and determinants addressed* (sun protection, healthy diet, and so on) *for population groups*.

Queensland's 2000-01 health promotion initiatives are classified by the *public health expenditure core category* of 'Selected health promotion', as used in national public health expenditure reporting,[61] while the (main) *function* or purpose is to 'Promote health and prevent disease, disability and injury' (using the public health functions developed in this project). Associated public health intervention *methods used* in the health promotion initiatives are also listed (e.g. intersectoral advocacy, community action). *Partnerships* and *stakeholders* are shown as test data.

This classification of a public health activity is much better than a one-dimensional classification at answering the questions listed in *Box 2* in *Section 3.1.3* as a practical test for the classification. For instance, in response to the question 'How much was spent last year on the prevention of obesity?', *Figure 8* shows that public health activities for which the *function* is 'prevention' and the *health issue* is 'obesity' can

---

[60] AIHW 2004b.

[61] AIHW 2004b: 56.

easily be identified, and the values in the 'expenditure' slot (attribute) for these activities can then be summed.

Example 2 Public health policies

A selection of public health policies compiled from publicly available documents accessible on the internet were classified using the top-level classes of the public health classification. An example public health policy is detailed in *Figure 9*.

Figure 9 Detail of a classified public health policy

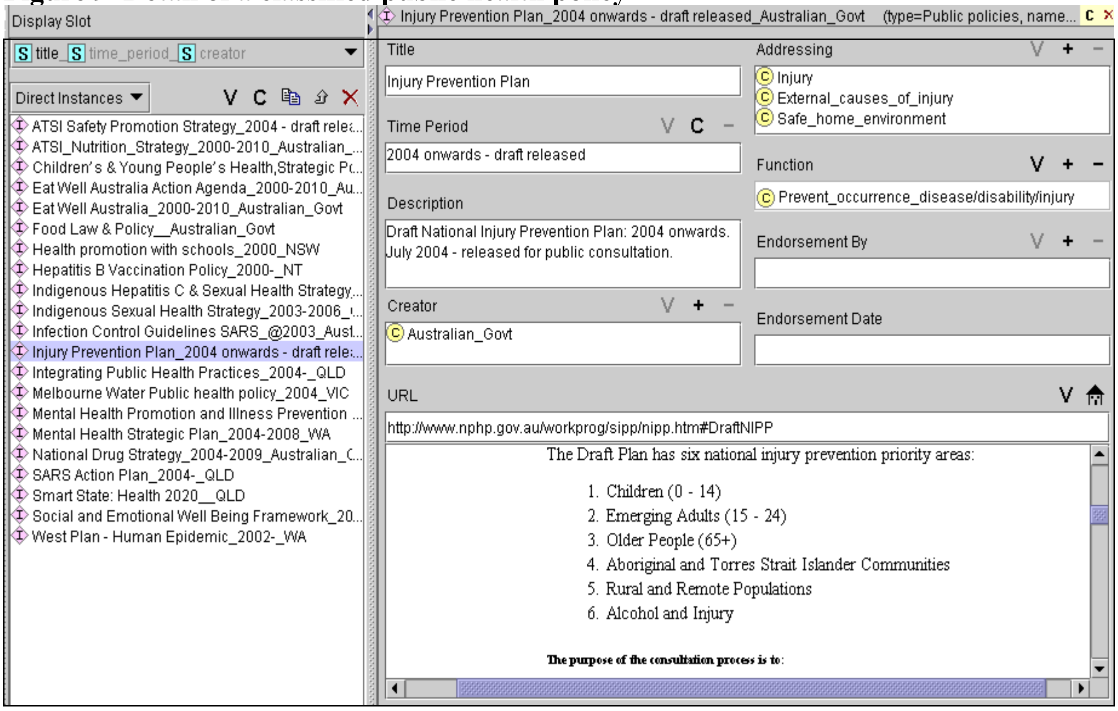

As previously, in *Figure 9*, the symbol denotes classes and subclasses, while the symbol denotes 'instances' or individual cases, for example, a particular public health policy. *Figure* 9 shows detail on the Australian Government Draft National Injury Prevention Plan (NPHP 2004) and the health issues it *addresses* (external causes of injury, safe home environment, and so on). The plan is assigned to the *function* subclass 'Prevent occurrence of disease, disability, and injury'. Capture of the *URL* for the published policy allows rapid access to the policy through the internet. Details of the jurisdictions and/or portfolios that have endorsed the plan can be captured in additional slots.

These examples do not completely illustrate the full power of a 'third generation' multi-dimensional classification for public health, developed using a formal ontology-building tool such as *Protégé*. While nothing can replace human knowledge and intelligence in the comprehensive collection, description (classification, indexing) and use of complex information, in the future it is envisaged that semantic tagging[62] of documentation and other written resources will allow much more *meaningful* information to be routinely made available to humans, through machine processing of

[62] HTML is an example of a lower level tagging language.

this ‘computable’ information. More information on this aspect of the project is presented in *Appendix D*.

3.2.5 Other classes considered

In addition to those top-level classes discussed in detail above, other potential classes were identified in the first round of development. These included:

Geography/access to health services (e.g. urban/rural/remote geographic classification).
Intervention target or focus (e.g. target population defined by age, sex, ethnicity) and intervention type.
Performance measures (e.g. the national health performance framework).
Precepts, principles, philosophy (e.g. equity).
Service production/provision (where service is produced/provided e.g. institutional health services, non-institutional health services) and service delivery/**settings** (where service is delivered e.g. school, workplace, community).
Sources of funds (e.g. health/non-health; levels of government).
Theories and models (e.g. ‘harm minimisation’, ‘user pays’).
Time (e.g. incubation periods, time-lags, investment periods, break-even points).
Workforce (e.g. public health specialists, local government workers, school nurses).

Potential classes that were identified in later consultations included:

Contextual/macro-environmental/ecological factors that affect but are outside the influence of public health (e.g. factors that would be picked up in environmental scanning).
Interventions as public health activities/strategies that are related but different to methods.
Outcomes, including outcome indicators and reporting (e.g. national public health system performance measures), necessary to ‘close the loop’ and complete the program logic for public health.
Policy including various views: policy development as an activity or ‘method’ or a cross-cutting component of all functions; policies as a class of things in existence (e.g. as in a policy register or library); policy as enhancing understanding of practice, cross-referenceable to other areas of interest.
Population groups as defined in terms of attributes and characteristics from other classes (e.g. age, sex).
Research/evidence allowing integration with the university sector, to link research and policy and practice, and to build the evidence base for public health.
Risk factors (part of the ‘determinants of health’ class).
Partners and stakeholders in the public health effort.

Although the project focussed on only a few selected classes, many of the other areas listed above were considered in detail. In some cases the topic area suggested has been captured in the broad structure (e.g. ‘settings’ have been included among the top-level classes). In other cases, the topic area has been built into the public health classification as attributes and characteristics of classes. Some are demonstrated in the examples of practical applications in *Section 3.2.4*. For instance, stakeholders and population groups are shown as attributes (slots) of ‘public health activities’ in *Example 1*. ‘Policy’ has been represented as a register or library of existing policies in *Example 2*. ‘Research’ should be identifiable through classification using the ‘methods’ class (which includes the subclass ‘research and evaluation methods’). ‘Workforce’ and ‘workforce development capacity’ have been included as subclasses of the ‘resources and infrastructure’ class, as has ‘time’.

3.2.6 Issues for further consideration

Definitional issues that were discussed during the project have been summarised as discussion points in *Box 2*. In *Box 3* a range of other issues, raised throughout this report, are summarised for the further consideration of public health experts.

Box 3 Classification of public health: summary of issues for further consideration

**Principles**	Is there agreement with the principles of development: multi-dimensional, inclusive rather than exclusive, broad rather than narrow?
**Scoping**	Should public health classification be restricted to a domain solely within health or should it include specific activities of other sectors (e.g. education, transport, local government) that have public health as a primary or secondary purpose (e.g. immunisation organised by local government)?
**Top-level classes**	Is there agreement on the top-level classes?
**Public health functions**	▪ Are the public health functions appropriate?<br>▪ Are all important functions captured (e.g. is quality assurance a public health function in Australia)?<br>▪ Is the division between primary and instrumental functions clear and useful?
**Subclasses**	Are there any important subclasses that are currently missing from the first two levels of the public health classification (see *Table* 7)?
**Properties and attributes**	What are the important characteristics of agreed top-level classes?

4 Next steps and recommendations

Phase one of the project has produced version one of a public health classification, and achieved a degree of consensus among Australian public health experts regarding its major classes, and their structure at the top levels. The classes of public health 'functions', 'determinants of health' and 'methods' of intervention have been identified as priorities for further development.

Many of the public health experts consulted during phase one of the project indicated that they were keen to continue their engagement. Most were positive about the project. They identified a range of practical applications for a public health classification that extended far beyond its uses for reporting public health activity and expenditure. The consultation process also brought to light a variety of issues—including areas of basic disagreement about the nature and boundaries of public health practice—that warrant more work. These are set out throughout this report in boxes.

It is proposed that the second phase of the project will further extend the availability of, and seek feedback on, the public health classification through a web-based version, and develop a proposal for its future development and support.

Because it attempts to capture the breadth of public health activity, and to serve multiple uses, the public health classification has a necessarily complex, multi-dimensional structure that is difficult to present adequately in paper-based forms. A web-based version, rendered in HTML, will allow interactive engagement and easier access to the structure, coverage and documentation (e.g. definitions). An early version of the classification was mounted on a test website and demonstrated in consultations with reasonable acceptance and understanding of its use as a navigation tool. A facility to collect structured feedback—rather than just adding large numbers of new classes and subclasses—and processes to compile and review this information will be needed to improve the utility of the classification for practical applications.

Developing a plan for the ongoing development and support of the classification will involve consideration of governance and maintenance arrangements, as well as the issues of access, availability and intellectual property ownership and management. Maintenance of classification systems can be difficult, time-consuming and thankless work. International classifications, like that of diseases, rely on a lengthy consensual process of experts to identify and agree upon new entries.[63] However, new capabilities made possible by the Internet and the development of the Semantic Web present opportunities to distribute the maintenance burden across many contributors, and to dramatically speed up consensual agreement.[64] These will be explored as part of scoping the requirements for ongoing development and support of the classification.

Further development of the classification will emphasise its relationships with classifications that are already in existence and widely used as standards. The public health classification, as it is currently structured, has subclasses that simply reference or point to relevant external classifications. These include (but are not limited to)

[63] See for instance, Bowker 1996.

[64] See Mathes (2004) on 'folksonomies', and Graeber et al. (2004), who describe the International Society for Neuropathology's adoption of an open-source or 'free community' approach to speed up and improve the development of the International Classification of Diseases of the Nervous System.

Australian standards (e.g. geographical, industry, and occupational classifications, other standards promulgated by AIHW and ABS) and the international classifications of diseases, functioning and disability, and external causes of injury[65] (see *Figure 7*). In a similar vein, it is proposed to investigate the possible inclusion of the public health classification in the set of standard classifications known as the Australian Family of Classifications.[66]

Recommendations

It is recommended that phase two of the Public Health Classifications Project should:

- Focus on further developing the classes of public health 'functions', 'determinants of health' and 'methods' of intervention;
- Develop and release a web-based version of the public health classification with facilities for eliciting structured feedback and managing contributions to the further development and refinement of the classification;
- Develop a plan for ongoing development, support and governance of the public health classification;
- Further specify links or relations between the public health classification and relevant existing classifications and standards (with due regard for intellectual property rights); and
- Investigate inclusion of the public health classification in the Australian Family of Classifications.

---

[65] WHO 1992-94, WHO 2001, and ICECI Coordination and Maintenance Group 2004.

[66] CTWG 2004.

Glossary

Class (noun)

A number of things regarded as forming one group through the possession of similar qualities; a kind; sort. (Delbridge & Bernard 1998)

Classes are the focus of most ontologies. They describe concepts in the domain. For example, the class of public health 'functions' represents all public health functions. Specific functions, for example, 'protect from threats to health', are instances of this class. A class can have subclasses that represent concepts that are more specific than the superclass. For example, we can divide the class of all public health 'functions' into 'assess...', 'protect...' and 'promote...' functions.[67] Alternatively, we can divide the class of all public health functions into primary and secondary functions. (Noy & McGuinness 2001: 3)

Class hierarchy

An arrangement of classes in a taxonomic (subclass–superclass) hierarchy. A class hierarchy represents an 'is-a' relation, where a class X is a subclass of A if every instance of X is also an instance of A. A class hierarchy thus represents a set of classes related by inheritance. A class hierarchy is typically shown as a tree structure for single inheritance or as a lattice structure for multiple inheritance (where nodes represent classes and are connected by arcs to indicate inheritance relations).

In an ontology there is no single correct class hierarchy for any given domain. The hierarchy depends on the possible uses of the ontology, the level of the detail that is necessary for the application, personal preferences, and sometimes requirements for compatibility with other models. (Noy & McGuinness 2001: 6-8)

Classification system

A system for classifying things; in a library, a system of arranging items according to broad fields of knowledge and specific subjects within each field. To classify means to arrange or distribute in classes; to place according to class.

Example: International Classification of Diseases (ICD) (WHO 1992-94).

Computable information

Computable information is information that can be readily manipulated and transformed by computers. Currently a great deal of information (on the Web and elsewhere) can be read by computers but not manipulated or understood by them. In the near future, the Semantic Web being developed by Sir Tim Berners-Lee, one of the founders of the World Wide Web, and others, will make information computable and connectable by adding semantic information, based on ontologies and classifications, to elements within text (Berners-Lee 2001).

Determinants of health

Determinants of health are factors that influence health status and determine health differentials or health inequalities. They are many and varied and include, for example, natural, biological factors, such as age, gender and ethnicity; behaviour and lifestyles, such as smoking, alcohol consumption, diet and physical exercise; the physical and social environment, including housing quality, the workplace and the wider urban and rural

---

[67] See *Section 3.2.2* for more information on the public health functions in a public health classification.

environment; and access to health care (Lalonde 1974, Labonté 1993). All of these are closely interlinked and differentials in their distribution often lead to health inequalities (WHO 1998a).

Dimension

A part or aspect of something. For example, one dimension of public health is the settings in which public health work is carried out. A dimension is a property or construct whereby aspects of something can be distinguished (e.g. public health settings can be distinguished from public health functions and from public health methods). A dimension can also be described as a group of similar things that are from the same category of information (e.g. home and workplace settings are part of the settings dimension). Hence multi-dimensional, to have many aspects or dimensions (e.g. to provide a unified framework for multiple public health uses, a *multi-dimensional* classification is needed).

Disease prevention—see also prevention, primary prevention

Disease prevention refers to measures taken to prevent the occurrence of disease, to arrest or slow its progress and to reduce its consequences. Examples of disease prevention measures include risk factor reduction, screening and early intervention.

Primary prevention of disease is directed towards preventing the initial occurrence of a disease. Secondary and tertiary prevention aim to arrest or slow the progression of existing disease and to reduce its effects through early detection of complications and appropriate treatment; or to reduce the occurrence of relapses and the establishment of chronic conditions through, for example, effective rehabilitation (WHO 1998a).

Function (noun)

The kind of action or activity proper to a person, thing, or institution (Delbridge & Bernard 1998: 452). The function, purpose, role or use of something; for example, the function of public health is 'to protect and promote health, and to prevent illness, injury and disability' (NPHP 1998).

Injury prevention—see prevention

'is-a' relation—see class hierarchy

Machine readable—see computable information, Semantic Web

Metadata

Information about data. Metadata can describe the fields and formats of databases and data warehouses, documents and document elements such as Web pages or research papers. Metadata management is a functional component of an information management architecture.

<u>Example</u>: the descriptive information provided in the 'META' tags in an HTML or XML document header that give information about the document.

Multi-dimensional—see dimension

Ontology

A model of a particular field of knowledge - the concepts relevant to that field (e.g. the field of public health), and their attributes, as well as the relationships between the concepts. In the

Protégé ontology development software,[68] an ontology is represented as a set of classes that have associated slots (attributes).

In philosophy, ontology describes a branch of metaphysics concerned with the nature and relations of being. The term has been redefined by the knowledge engineering and artificial intelligence communities to refer to a formalised description of the concepts and relationships that exist within a specific domain and all that can be represented about that domain. Ontologies can be mental models, computer models, or a combination of both. Ontologies provide a means by which characteristics of a specific representation can be assumed and behaviour predefined (Kemp & Vckovski 1998). Multiple user views can be accommodated by providing translations between different ontologies.

An ontology defines a common vocabulary for researchers who need to share information in a domain. It includes machine-interpretable definitions of basic concepts in the domain and relations among them (Noy & McGuinness 2001).

Ontologies are developed for the purposes of:

- Sharing common understanding of the structure of information among people or software agents,
- Enabling re-use of domain knowledge,
- Making domain assumptions explicit,
- Separating domain knowledge from operational knowledge, and
- Analysing domain knowledge.

Example: The (US) National Library of Medicine's Unified Medical Language System (UMLS) 'knowledge sources' and associated lexical programs for system developers. The Meta-thesaurus is organised by concept or meaning. Its purpose is to link alternative names and views of the same concept together and to identify useful relationships between different concepts.

Population health

Organised efforts focused on the health of defined populations in order to promote and maintain or restore health, to reduce the amount of disease, premature death and discomfort and disability due to disease. Programs, services and institutions here emphasize the prevention of disease and the health needs of the population as a whole. Among a broad scope of disciplines, various knowledge and skills are used, such as bio-statistics, epidemiology, planning, organisation, management, financing and evaluation of health programs, environmental health, application of social and behavioural factors in health and disease, health promotion, health education and nutrition. (IIME 2002)

Preventable conditions

Preventable conditions include many chronic, non-communicable diseases such as cardio-vascular disease, type 2 diabetes, obesity, chronic lung disease; conditions amenable to early detection and treatment such as breast and cervical cancer, high blood pressure; communicable diseases such as HIV/AIDS, food borne illness, vector borne diseases, vaccine preventable diseases; intentional and unintentional injuries; many mental health problems and related conditions such as substance abuse and family dysfunction. (Straton & Sindall 2001: 1)

[68] *Protégé* is developed by Stanford University, see http://protege.stanford.edu.

Prevention

Prevention is characterised by activities that are taken to reduce the possibility that something will happen, or to minimise harm if it does occur. The prevention of illness or disability requires the identification of the factors that contribute to poor health and modifying, reducing or eliminating them, or, conversely, building and strengthening *protective* factors. Prevention is usually taken as a core responsibility of organised health systems—alongside the curative, restorative and palliative functions—and is a key element in achieving health improvement and the reduction of the burden of disease in society. Prevention is also an important component of many other branches of social policy (for example crime prevention, child abuse prevention), many of which also contribute, directly or indirectly, to health.

It has been customary to categorise prevention at different levels, in terms of primary, secondary and tertiary prevention. Thus the goal of *primary prevention* is reducing the incidence of disease by preventing its occurrence, *secondary prevention* aims to prevent progression of disease though early detection, usually by screening at an asymptomatic stage and early intervention,[69] and the goal of *tertiary prevention* includes minimisation of the impact of established disease, and prevention of complications and further disability through effective treatment and rehabilitation. While the terminology used can vary in different fields (for example a slightly different set of categories is often used in relation to mental health[70]), the basic concepts and objectives of prevention are essentially the same.

It is often useful to think in terms of a hierarchy or spectrum of objectives for preventive activity, aimed at different points on the causal pathway, and for which there is often an important time dimension. For example, the short term aim of a preventive intervention at a certain point in time may be to change beliefs in the community about the risks of smoking; the intermediate objective may be to reduce uptake of smoking and smoking prevalence and the long term goal a reduction in rates of coronary heart disease and lung cancer. (Straton & Sindall 2001: 1)

Prevention and public health services in the OECD System of Health Accounts

Prevention and public health services comprise services designed to enhance the health status of the population as distinct from the curative services, which repair health dysfunction. Typical services are vaccination campaigns and programmes. (OECD 2000: 121)

Primary prevention—see also prevention, disease prevention

Primary prevention refers to the protection of health by personal and community wide effects, such as preserving good nutritional status, physical fitness, and emotional well-being, immunising against infectious diseases, and making the environment safe. There are no precise boundaries between the primary, secondary and tertiary levels of prevention. (IIME 2002)

[69] A notable exception to this use of the term is found in the area of cardiovascular disease prevention and control where secondary prevention is commonly used to refer to prevention of a second heart attack.

[70] In the mental health field primary prevention is further divided into approaches designated as universal, selective or indicated prevention, depending on whether they are applied to the whole population (universal) or sub-groups (selective) or those at an early stage of risk (indicated). A similar approach was used by the AIHW in development of the indicator framework for monitoring the National Health Priority Areas.

Public health

Public health is the organised response by society to protect and promote health, and to prevent illness, injury and disability. The starting point for identifying public health issues, problems and priorities, and for designing and implementing interventions, is the population as a whole, or population sub-groups. (NPHP 1998)

Although priorities may change as technology advances and social values change, the public health goals of reducing the level of disease, the risk of premature death and disease-produced discomfort and disability in the population remain the same. Public health hazards may be environmental, nutritional, related to alcohol or other drugs, food safety, communicable and non-communicable diseases, and other hazards causing injury. (NPHP 1998)

Public health activities

Public health activities and processes can be grouped into three key areas: intelligence, interventions, and infrastructure (NPHP 1998).

> **Public health intelligence** is involved with gathering and analysing information about the determinants of health, the causes of ill health and the patterns and trends of health and ill health in the population.
>
> **Public health intervention** refers to developing policy, setting priorities for actions, developing plans, coordinating services, strategies and interventions aimed at prevention, protection and promotion of the health of the community, where promotion is the action taken to solve public health problems.
>
> **Public health infrastructure** refers to the administrative, legislative and informational systems developed for making priorities, for developing policy, for funding, for monitoring and surveillance, for research and evaluation, for program delivery, and includes the workforce required to accomplish these tasks. (NPHP 1998)

Government-funded public health activity is described as an important part of the Australian health care system, with public health activities generally representing the organised response of society to protect and promote the current and future health of the whole population or of specific subgroups of the population, which can be viewed as a form of investment in the overall health status of the nation. (AIHW 2004b: 1)

Public health core functions

The nine public health core functions promulgated by the National Public Health Partnership (NPHP 1998) are:

1. Assess, analyse and communicate population health needs and community expectations
2. Prevent and control communicable and non-communicable diseases and injuries through risk factor reduction, education, screening, immunisation and other interventions
3. Promote and support healthy lifestyles and behaviours through action with individuals, families, communities and wider society
4. Promote, develop and support healthy public policy, including legislation, regulation and fiscal measures
5. Plan, fund, manage and evaluate health gain and capacity building programmes designed to achieve measurable improvements in health status, and to strengthen skills, competencies, systems and infrastructure
6. Strengthen communities and build social capital through consultation, participation and empowerment
7. Promote, develop, support and initiate actions which ensure safe and healthy environments

8. Promote, develop and support healthy growth and development throughout all life stages
9. Promote, develop and support actions to improve the health status of Aboriginal and Torres Strait Islander people and other vulnerable groups.

Public health defined by WHO

Public health has been defined by the World Health Organization as 'the art of applying science in the context of politics so as to reduce inequalities in health while ensuring the best health for the greatest number' (WHO 1998a cited in WHO 2003: 1).

Public health expenditure reporting: core public health activities

The core public health activities in public health expenditure reporting are defined as 'nine types of activities undertaken or funded by the key jurisdictional health departments that address issues related to populations, rather than individuals. Does not include treatment services.' (AIHW 2004b: 145)

Government-funded public health activity is described as an important part of the Australian health care system, with public health activities generally representing the organised response of society to protect and promote the current and future health of the whole population or of specific subgroups of the population, which can be viewed as a form of investment in the overall health status of the nation. (AIHW 2004b: 1)

Public health medicine

Public health medicine is that branch of medical practice that is primarily concerned with the health and care of populations. It is concerned with the promotion of health and the prevention of disease and illness; the assessment of a community's health needs; and the provision of services to communities in general and to specific groups within them. (AFPHM 2002a)

Public health research

Research involving communities or populations, typically outside health care institutions, undertaken to identify the factors which contribute to ill-health in populations and ways of influencing these factors to prevent disease. It includes epidemiology, social and behavioural sciences, health services research on population-based health interventions, and evaluating the efficacy and effectiveness of preventive measures. (HMRSR 1998: A6.4, Saracci 2004: 240)

Public health workforce

The public health workforce is defined as those involved in protecting, promoting and/or restoring the collective health of whole or specific populations (as distinct from activities directed to the care of sick or frail individuals). (Rotem et al. 1995 cited in Riddout et al.2002: 19).

Resource Description Framework

Resource Description Framework (RDF) 'is a foundation for processing metadata; it provides interoperability between applications that exchange machine-interpretable information on the Web. RDF emphasizes facilities to enable automated processing of Web resources. RDF can be used in a variety of application areas; for example: in resource discovery to provide better search engine capabilities, in cataloguing for describing the content and content relationships available at a particular Web site, page, or digital library, by intelligent software agents to facilitate knowledge sharing and exchange, in content rating, in describing collections of pages that represent a single logical "document", for describing intellectual property rights of Web pages, and for expressing the privacy preferences of a user as well as the privacy

policies of a Web site. RDF with digital signatures will be key to building the "Web of Trust" for electronic commerce, collaboration, and other applications' (W3C 1999).

The Semantic Web Environmental Directory describes RDF as the 'equivalent of the language for writing Web pages, HTML (HyperText Markup Language), for the Semantic Web. The Semantic Web uses RDF as the basic language for representing metadata about any kind of resource on the Web' (SWED undated).

Secondary prevention—see also Prevention

Secondary prevention can be defined as the measures available to individuals and populations for the early detection and prompt and effective intervention to correct departures from good health. There are no precise boundaries between primary, secondary and tertiary levels of prevention. (IIME 2002)

Semantic Web

The Semantic Web provides a common framework that allows data to be shared and reused across application, enterprise, and community boundaries. It is a collaborative effort led by W3C with participation from a large number of researchers and industrial partners. It is based on the Resource Description Framework (RDF), which integrates a variety of applications using XML for syntax and URLs for naming.

'The Semantic Web is an extension of the current web in which information is given well-defined meaning, better enabling computers and people to work in cooperation' (Berners-Lee et al. 2001). The Semantic Web and computable information are the visions of Tim Berners-Lee, the creator of the World Wide Web (familiar to us through Google[71] and other search engines), who views this future Web as a web of data, 'like a global database', where 'information is given well-defined meaning, better enabling computers and people to work in cooperation'. Making information on the Web 'semantic' (or meaningful) means much more efficient searching 'as though it were one giant database, rather than one giant book' (Berners-Lee 1998).

The infrastructure of the Semantic Web will allow machines as well as humans to make deductions and organise information. The approach is to develop languages that express information in machine processable forms. The architectural components include semantics (meaning of elements), structure (organisation of elements), and syntax (communication). Abstract representation of data is being based on existing standards (eg RDF – Resource Description Framework) and standards yet to be defined, and is in development by the World Wide Web Consortium (W3C), in collaboration with researchers and industrial partners. (Berners-Lee 1998, Berners-Lee et al. 2001, Berners-Lee & Miller 2002)

Slot

In an ontology, slots describe properties of each concept describing various features and attributes of the concept (sometimes called roles or properties). (Noy & McGuinness 2001: 8)

Subclass—see class

Taxonomy

A classification, especially in relation to its principles or laws; the department of science/s that deal with classification. A taxonomy is hierarchical, with the higher levels being larger, more inclusive and broadly defined, while the lower levels are more restrictive and specific.

---

[71] An example of a 'Semantic Web Ontology …' is *Swoogle* (http://swoogle.umbc.edu/index.php University of Maryland, BC).

Example: the classification of plant and animal life into natural, related groups in descending order: phylum, class, order, family, genus, species.

Terminology

The system of terms belonging to a science, art, or subject; nomenclature.

A controlled vocabulary contains metadata about terminology to make it easier to search and maintain knowledge management systems that integrate information from multiple sources and applications.

Example: SNOMED CT® - Systematized Nomenclature of Medicine-Clinical Terms (produced by the College of American Pathologists) is a comprehensive clinical terminology, and one of a suite of designated standards for use in US Federal Government systems for the electronic exchange of clinical health information, and is being implemented throughout the National Health Service in the UK.

Tertiary prevention—see also Prevention

Tertiary prevention consists of the measures available to reduce or eliminate long-term impairments and disabilities, minimize suffering caused by existing departures from good health, and to promote the patient's adjustment to irremediable conditions. This extends the concept of prevention into the field of rehabilitation. There are no precise boundaries between primary, secondary and tertiary levels of prevention. (IIME 2002)

Thesaurus

A storehouse or repository, as of words or knowledge; a dictionary, encyclopedia or the like, especially a dictionary of synonyms and antonyms.

Technical thesauri are used in search-language normalisation as they specify terms to be used (preferred terms), broader and narrower terms in the hierarchy, as well as related terms (non-hierarchically related, e.g. antonyms) and non-preferred terms (synonyms for the preferred term).

Example: MeSH (Medical Subject Headings) – the (US) National Library of Medicine's controlled vocabulary, used to index articles for MEDLINE and PubMed. MeSH terminology provides a consistent way to retrieve information that uses different terminology for the same concepts.

Wicked problem

The **wicked problem** concept was originally proposed by Rittel and Webber (1984) in the context of social planning. They pointed out that in solving a wicked problem, the solution of one aspect may reveal another, more complex problem. Ten rules define the form of a wicked problem, including:

1. There is no definitive formulation of a wicked problem.
2. Wicked problems have no stopping rule.
3. Solutions to wicked problems are not true-or-false, but good-or-bad.

Every wicked problem is essentially unique, and can be considered to be a symptom of another problem. (The last rule is that: The planner (designer) has no right to be wrong.)

References and sources

Abelson P, Taylor R, Butler J, Gadiel D, Clements M, Mui S-L (2003) *Returns on investment in public health*: An epidemiological and economic analysis prepared for the Department of Health and Ageing. Canberra: DoHA. http://www.health.gov.au/pubhlth/publicat/document/roi_eea.pdf accessed 3 October 2004

Association of Schools of Public Health (ASPH) (2004) What is Public Health? http://www.asph.org/document.cfm?page=300 accessed 27 July 2004.

Australasian Faculty of Public Health Medicine (AFPHM) (2002a) Competency Areas in Public Health Medicine. Sydney: AFPHM. http://www.racp.edu.au/afphm/advtrain/competency.htm accessed 14 July 2004.

Australasian Faculty of Public Health Medicine (AFPHM) (2002b) What is Public Health Medicine? Sydney: AFPHM. http://www.racp.edu.au/afphm/defn.htm accessed 14 July 2004.

Australian Government Department of Health and Ageing (DoHA) (2004) The National Public Health Partnership. http://www.health.gov.au/pubhlth/about/nphp.htm accessed 2 August 2004.

Australian Institute of Health and Welfare (AIHW) (1999) *National Public Health Information Development Plan 1999*. [Jointly prepared by AIHW and the National Public Health Information Working Group (NPIWG.] AIHW Cat. No. HWI-22. Canberra: AIHW. http://www.aihw.gov.au/publications/health/nphidp99/ accessed 2 August 2004.

Australian Institute of Health and Welfare (AIHW) (2003) *Health expenditure Australia 2001–02*. Health and Welfare Expenditure Series no. 17. AIHW Cat. no. HWE 24. Canberra: AIHW. http://www.aihw.gov.au/publications/index.cfm/title/9307 accessed 5 July 2004.

Australian Institute of Health and Welfare (AIHW) (2004a) Expenditure: Public Health. http://www.aihw.gov.au/expenditure/public_health.html accessed 6 July 2004.

Australian Institute of Health and Welfare (AIHW) (2004b) *National public health expenditure report 2000–01*. Health and Welfare Expenditure Series no. HWE 25. Canberra: AIHW. http://www.aihw.gov.au/publications/index.cfm/title/10012 accessed 5 July 2004.

Australian Institute of Health and Welfare (AIHW) (2004c) *Health system expenditure on disease and injury in Australia, 2000–01*. Health and Welfare Expenditure Series no. HWE 19. Canberra: AIHW. http://www.aihw.gov.au/publications/index.cfm/title/10012 accessed 5 July 2004.

Beck T (1999b) *Using Gender-Sensitive Indicators*: A Reference Manual for Governments and Other Stakeholders. London: Commonwealth Secretariat.

Beck T and Stelcner M (1997) *Guide to Gender Sensitive Indicators*. Hull: CIDA (Canadian International Development Agency).

Bennett J (2003) *Investment in Population Health in Five OECD Countries*. OECD Health Working Papers No. 2. Paris: OECD. http://www.oecd.org/dataoecd/30/39/2510907.pdf accessed 3 October 2003.

Berman P (1999) 'What can the U.S. Learn from National Health Accounting Elsewhere?' *Health Care Financing Review*, 21(2): 47-632.

Berners-Lee T (1998) A roadmap to the semantic web. World Wide Web Consortium (W3C). http://www.w3.org/DesignIssues/Semantic.html accessed 4 February 2005.

Berners-Lee T and Miller E (2002) 'The Semantic Web lifts off', *ERCIM News* (51): 9-11. http://www.ercim.org/publication/Ercim_News/enw51/berners-lee.html accessed 4 February 2005.

Berners-Lee T, Hendler J and Lassila O (2001) 'The Semantic Web', *Scientific American* 284(5): 34-43. http://www.scientificamerican.com/article.cfm?articleID=00048144-10D2-1C70-84A9809EC588EF21&pageNumber=1&catID=2 accessed 4 February 2005.

Bowker GC (1996) 'The history of information infrastructures: the case of the International Classification of Diseases', *Information Processing and Management*, 32(1): 49-61.

http://www.ics.uci.edu/~corps/phaseii/Bowker-IntlClassDiseases-IPM.pdf accessed 2 June 2005.

Browne E and May L (2004) Appendix A - Stream 1: Clinical Information Framework. [?Canberra: DoHA]. http://www.health.gov.au/healthconnect/pdf_docs/cip_pdfs/cipp1aa.pdf accessed 28 September 2004.

Buckett K and Hunter J (2004) 'Public health and the SA health reform agenda', *Public Health Bulletin South Australia* (1): 1-2. http://www.dh.sa.gov.au/pehs/publications/public-health-bulletin.htm accessed 11 January 2005.

Chief Medical Officer, United Kingdom (CMO UK) (2003) *Public health in England*. London: UK Dept of Health. http://www.dh.gov.uk/AboutUs/MinistersAndDepartmentLeaders/Chief-MedicalOfficer/Features/FeaturesBrowsableDocument/fs/en?CONTENT_ID=4102835&MULTIPAGE_ID=5017848&chk=9GNrJZ accessed 5 September 2005.

Classifications and Terminologies Working Group (CTWG) of the [Australian] National Health Information Group (2004) *The Australian Family of Health and Related Classifications*. Canberra: AIHW. http://www.aihw.gov.au/committees/ctwg/public_resources/princ_australia_300802.doc accessed 2 August 2005.

Commonwealth Grants Commission (CGA) (2002) *Population and Preventive Health*. Discussion Paper CGC 2002/231, prepared for the Commission Staff Conference November 2002. Canberra: CGC.

Delbridge A and Bernard JRL (Eds) (1998) *The Macquarie Concise Dictionary*. 3rd ed. Sydney: Macquarie Library.

Deming WE (1993) *The New Economics for Industry, Government, Education* (2nd ed.). Cambridge, MA: Massachusetts Institute of Technology, Center for Advanced Engineering Study.

Fraser J (2005) 'Population and public health in Australian general practice: Changes, challenges and opportunities', *Australian Family Physician*, 34(3): 177-179.

Frauenfelder M (2004) 'Connecting the dots', *Sydney Morning Herald*, Oct 19, 2004, http://www.smh.com.au/articles/2004/10/18/1097951617438.html accessed 10 November 2004.

Graeber MB, Lowe J, Radotra B (2004) 'A free community approach to classifying disease', PLoS Medicine, 1(2): e16.Gruber TA (1993) 'A Translation Approach to Portable Ontology Specifications', Knowledge Acquisition 5(2): 199-220. http://medicine.plosjournals.org/archive/1549-1676/1/2/pdf/10.1371_journal.pmed.0010016-L.pdf accessed 3 June 2005.

Gruninger M and Fox MS (1995) 'Methodology for the Design and Evaluation of Ontologies', Workshop on Basic Ontological Issues in Knowledge Sharing, International Joint Conference on Artificial Intelligence (IJCAI), 1995, Montreal. http://www.eil.utoronto.ca/enterprise-modelling/papers/gruninger-ijcai95.pdf accessed 12 March 2005.

Hayes M and Dunn JR (1998) *Population health in Canada: systematic review*. CPRN Study No. H 01. CIHI. Hayes & Dunn 1998. http://secure.cihi.ca/cihiweb/dispPage.jsp?cw_page=cphi_-aboutph_e accessed 27 February 2005.

Health and Medical Research Strategic Review (HMRSR) (1998) *The virtuous cycle: working together for health and medical research*. [Discussion document] Canberra: Commonwealth of Australia.

Hyde J (1999) Recognising Social Health. [publication details]

ICECI Coordination and Maintenance Group (2004) International Classification of External Causes of Injuries (ICECI) (version 1.2). Amsterdam: Consumer Safety Institute, and Adelaide: AIHW National Injury Surveillance Unit..

Institute for International Medical Education (IIME) (2002) *Glossary of Medical Education Terms*. White Plains, New York: IIME. http://www.iime.org/glossary.htm accessed 7 July.

Institute of Medicine (IOM), Committee for the Study of the Future of Public Health, Division of Health Care Services (1988) *The Future of Public Health.* Washington, DC: National Academy Press

International Organization for Standardization (ISO) (1988) *Documentation and information*. ISO Standards Handbook 1, 3rd ed. Geneva: ISO.

Kemp KK and Vckovski A (1998) 'Towards an ontology of fields', Proceedings of the Third International Conference on GeoComputation, 17-19th September 1998, Bristol, UK. http://www.institute.redlands.edu/kemp/papers/geocomputation.html accessed 11 February 2005.

Khaleghian P and Das Gupta M (2004) *Public Management and the Essential Public Health Functions*. World Bank Policy Research Working Paper 3220. Washington, DC: The World Bank Group. http://econ.worldbank.org/files/33192_wps3220.pdf accessed 26 July 2004.

Lalonde M (1974) *A new perspective on the health of Canadians*: A working document. Ottawa: Government of Canada. [republished 1981]

Lamar Soutter Library, University of Massachusetts Medical School (date) Evidence-based Practice for Public Health Project. http://library.umassmed.edu/ebpph/ accessed 7 July 2004.

Last JM (1987) *Public Health and Human Ecology*. Connecticut: Appleton & Lange.

Last JM (ed.) (2001) *A dictionary of epidemiology*. 4th ed. Oxford: Oxford University Press.

Latin America and Caribbean Regional Health Sector Reform Initiative (LACHSR) (2004) *Health Sector Reform Thesaurus*. http://www.lachsr.org/en/thesaurus/00000228.htm accessed 27 July 2004.

Mathers C, Vos T and Stevenson C (1999) *The Burden of Disease and Injury in Australia*. Cat. No. PHE 18. Canberra: AIHW.

Mathes A (2004) 'Folksonomies – Cooperative classification and communication through shared metadata'. [Graduate School of Library and Information Science, University of Illinois.] http://www.adammathes.com/academic/computer-mediated-communication/folksonomies.html accessed 11 January 2005.

Murray CJL and Lopez AD (1996) *The Global Burden of Disease: A Comprehensive Assessment of Mortality and Disability from Diseases, Injuries, and Risk Factors in 1990 and Projected to 2020* (Global Burden of Disease and Injury, Executive Summary of Vol 1). Boston: Harvard University Press. http://www.hsph.harvard.edu/organizations/bdu/GBDseries.html accessed 26 July 2004.

National Advisory Committee on SARS and Public Health (2003) *Learning from SARS: Renewal of Public Health in Canada*. Ottawa: Health Canada. http://www.hc-sc.gc.ca/english/protection/warnings/sars/learning.html accessed 28 July 2004.

National Health Performance Committee (NHPC) (2001) *National Health Performance Framework Report*. Brisbane: Queensland Health. http://www.health.qld.gov.au/nathlthrpt/framework.asp accessed 15 July 2003.

National Health Performance Committee (NHPC) (2002). *National Report on Health Sector Performance Indicators 2001*. Brisbane: Queensland Health. http://www.health.qld.gov.au/nathlthrpt/performance.asp accessed 15 July 2003.

National Health Performance Committee (NHPC) (2004) *National Report on Health Sector Performance Indicators 2003*. Canberra: AIHW. http://www.aihw.gov.au/publications/index.cfm/title/10085 accessed 2 March 2005.

National Public Health Partnership (NPHP) (1998) *Public Health in Australia: The Public Health Landscape: person, society, environment*. Melbourne: NPHP. http://hna.ffh.vic.gov.au/nphp/publications/broch/contents.htm accessed 3 August 2004.

National Public Health Partnership (NPHP) (2000a) *Public Health Practice in Australia today: A statement of core functions*. Melbourne: NPHP. http://www.nphp.gov.au/workprog/phpractice/corefuncts.htm, accessed 22 June 2004.

National Public Health Partnership (NPHP) (2000b) *A Planning Framework for Public Health Practice*. Melbourne: NPHP. http://www.nphp.gov.au/publications/phpractice/planfrwk.pdf accessed 2 August 2004.

National Public Health Partnership (NPHP) (2001) *Preventing Chronic Disease: A Strategic Framework*. Background Paper. Melbourne: NPHP. http://www.nphp.gov.au/publications-strategies/chrondis-bgpaper.pdf accessed 2 August 2004.

Noy NF and McGuinness DL (2001) 'Ontology development 101: A guide to creating your first ontology' [Knowledge Systems Laboratory, Stanford University, CA] http://protege.stanford.-edu/publications/ontology_development/ontology101.pdf accessed 4 February 2005.

NSW Health, Public Health Division (2000) *Healthy People 2005 – New Directions for Public Health in NSW*. Sydney: NSW Health Department. http://www.health.nsw.gov.au/health-public-affairs/publications/healthyppl/index.html accessed 8 July 2004.

Organisation for Economic Co-operation and Development (OECD) (2000) *A System of National Health Accounts*. Geneva: OECD.

Organisation for Economic Co-operation and Development (OECD) (2000) *International Classification of Health Accounts*. Geneva: OECD.

Owen T and Jorm L. (2002) *Public Health Performance Project: Report to the National Public Health Partnership Group*. Melbourne: National Public Health Partnership. http://www.nphp.gov.au/publications/phpractice/phpractice_project/findingsandrecs.pdf accessed 2 August 2004.

Pan American Health Organization (PAHO) (2002) *Public Health in the Americas: conceptual renewal, performance assessment, and bases for action*. Washington, DC: PAHO.

Public Health Classifications Project (PHCP) (2004) *Public Health Classifications Project: Background paper*. Melbourne: National Public Health Partnership. http://www.nphp.gov.au/workprog/phi/documents/phcpbkgrpaper.pdf accessed 7 January 2005.

Raphael D and Bryant T (2002) 'The limitations of population health as a model for a new public health', *Health Promotion International*, 17(2): 189-199.

Riddout L, Gadiel D, Cook K, Wise M (2002) *Planning framework for the Public Health workforce: Discussion Paper*. Melbourne: National Public Health Partnership. www.dhs.vic.gov.au/nphp/publications/wfpapers/wfplanning.pdf accessed 24 June 2004.

Rittel HW and Webber MM (1984) 'Planning problems are wicked problems' in Cross N (Ed) *Developments in Design Methodology*. New York: John Wiley.

Rotem A, O'Connor K, Bauman A, Black D, Dewdney J, Hodgkinson A. (1995) *The public health workforce education and training study*. Canberra: AGPS.

Royal Australian College of General Practitioners (RACGP) (2004) *Smoking, Nutrition, Alcohol and Physical activity (SNAP): A population health guide to behavioural risk factors in general practice*. South Melbourne, Vic: RACGP. http://www.racgp.org.au/guidelines/snap/ accessed 7 February 2005.

Saracci R (2004) 'Public health and epidemiological research: a blind spot among the European Union priorities?', *International Journal of Epidemiology*, 33(2): 240-242.

Semantic Web Environmental Directory (SWED) [undated] *SWED Glossary*. Bristol, UK: SWAD (Semantic Web Advanced Development)-Europe. http://www.swed.org.uk/swed/about/swed_glossary.htm#rdf accessed 14 February 2005.

Straton J and Sindall C (2001) 'The scope of prevention', [internal discussion paper prepared for the Australian Government Department of Health and Ageing, Population Health Division, National Centre for Disease Control.]

Straton J and Sindall C (2003) 'Perspectives on prevention terminology', paper prepared for the National Health Priority Action Council, May 2003. [Australian Government Department of Health and Ageing, Population Health Division, Strategic Planning Branch.]

Turrell G and Mathers CD (2000) 'Socioeconomic Status and Health in Australia', *Medical Journal of Australia*, Vol. 172, pp. 434- 438.

Turrell G, Oldenburg B, McGuffog I, Dent R (1999). *Socioeconomic determinants of health: towards a national research program and a policy and intervention agenda*. Brisbane: Queensland University of Technology.

US Department of Health and Human Services (DHHS), Public Health Functions Steering Committee (1995) *Public Health in America*. Washington, DC: US DHHS.

US Department of Health and Human Services (DHHS), Public Health Service (date) The Public Health Workforce, Report of the Public Health Functions Project. [no publication details]

Wikimedia Foundation (2005) Wikipedia. http://en.wikipedia.org/wiki/Main_Page accessed 27 April 2005.

World Health Organization (WHO) (1992-94) *International Statistical Classification of Diseases and Related Health Problems* (10th revision, vols. 1-3) (ICD-10). Geneva: WHO.

World Health Organization (WHO) (1998a) *Health Promotion Glossary*. WHO/HPR/HEP/98.1 Geneva: WHO.

World Health Organization (WHO) (1998b) *The World Health Report 1998: Life in the 21st Century – A vision for All*. Geneva: WHO.

World Health Organization (WHO) (2001) *International Classification of Functioning, Disability and Health (ICF)*. Geneva: WHO.

World Health Organization (WHO) (2004) Family of International Classifications: definition, scope and purpose.. http://www.who.int/classifications/icd/docs/en/WHOFICFamily.pdf accessed 2 August 2005.

World Health Organization (WHO), Regional Office for the Western Pacific (2003) *Essential public health functions: A three-country study in the Western Pacific Region*. Manila, Philippines: WHO, Regional Office for the Western Pacific. www.wpro.who.int/pdf/pub/unit_hsd/essential-_public_health_functions/essential_public_health_functions.pdf accessed 24 June 2004.

World Wide Web Consortium (W3C) (1999) Resource Description Framework (RDF) Model and Syntax Specification. Cambridge, MA: W3C. http://www.w3.org/TR/1999/REC-rdf-syntax-19990222/ accessed 3 March 2005.

Yach, D (1996) 'Redefining the scope of public health beyond the year 2000', *Current Issues in Public Health*, 2:247-252.

Acronyms and abbreviations

ABS	Australian Bureau of Statistics
AFC	Australian Family of Classifications
AIHW	Australian Institute of Health and Welfare
DoHA	Australian Government Department of Health and Ageing
ERPHO	Eastern Region Public Health Observatory
GPs	General Practitioners
HDA	Health Development Authority
HTML	Hyper Test Markup Language
ICD	International Classification of Diseases
ICD10	International Classification of Diseases, version 10
MESH	Medical Subject Headings
NGOs	Non-Government Organisations
NHA	National Health Accounts
NPHIWG	National Public Health Information Working Group
NPHL	National Public Health Language
NPHP	National Public Health Partnership (‘the Partnership’)
NPHPG	National Public Health Partnership Group
Partnership, the	National Public Health Partnership (NPHP)
OECD	Organisation for Economic Co-operation and Development
PHIDU	Public Health Information Development Unit
PHITS	Public Health Information Tagging Standard
PHOs	Public Health Observatories
PHPP	Public Health Performance Project
RACGP	Royal Australian College of General Practitioners
RDF	Resource Description Framework
SARS	Severe Acute Respiratory Syndrome
SHA	System of Health Accounts
SNOMED	Systematized Nomenclature of Medicine
STIs	Sexually Transmitted Infections
UMLS	Unified Medical Language System
URLs	Uniform Resource Locators
VicHealth	Victorian Health Promotion Foundation
W3C	World Wide Web Consortium
WHO	World Health Organization
XML	Extensible Markup Language

Appendices

Appendix A: Project inception and terms of reference

The Public Health Performance Project (2002) recommended the development of a classification system for public health that builds on the public health core functions and can be used for expenditure and performance monitoring. The Public Health Information Development Unit (PHIDU) carried out preliminary work and prepared a project proposal. The proposal for a seven-month project was endorsed by the National Public Health Information Working Group and funded by the National Public Health Partnership Group (NPHPG) in 2004.

The project objectives are to develop and endorse a higher-level classification that captures the full scope and breadth of public health activity in Australia, provides a unified framework for further developing public health performance indicators and categorising public health expenditure, and can be used to incorporate public health-relevant categories in 'classification of expenditure use' schemas.

Objective: to develop and endorse a higher-level classification that captures the breadth and scope of public health activity and provides a unified framework for multiple uses.

The NPHPG has listed the Public Health Classifications Project among its priorities for the next three years.

The project commenced in June 2004 and has been guided by a Reference Group that meets monthly on average, by teleconference, and provides advice and assistance to the Project Officer out-of-session as required. The Terms of Reference follow.

NATIONAL PUBLIC HEALTH PARTNERSHIP

Public Health Classifications Project Reference Group

Terms of Reference

Name

The Reference group is known as the 'Public Health Classifications Project Reference Group'.

Purpose

The purpose of the Reference Group is to:

1. Assist in developing a detailed operational plan for the project.
2. Provide comment on draft working papers and reports produced as part of the project.
3. Liaise with other stakeholders to inform them about the project, and seek their input as appropriate.
4. Recommend mechanisms for implementing and further developing the classifications developed by the project.
5. Endorse the final report of the project prior to its submission to the National Public Health Information Working Group.

Appointment to the Reference Group

The National Public Health Partnership Group and National Public Health Information Working Group nominates members of the Reference group.

Structure and reporting

The Reference group reports to the National Public Health Partnership Group through the National Public Health Information Working Group.

Secretariat

The secretariat comprises the Project Officer.

Meetings

The Reference Group will meet by teleconference monthly from April to September 2004, on dates set by the chair. Meetings will last for up to one hour.

Out-of-session material

The Reference group can be asked to comment on out-of-session material at the discretion of the chair. Material may be forwarded to members either in hard copy or by electronic mail.

Appendix B: Acknowledgement of contributors

Deriving a conceptual model of public health for the purpose of developing a unified, high-level classification that is useful for a range of applications has necessarily been a collaborative process. The Public Health Classifications Project Reference Group has led the development, and a range of interested public health experts in a sample of jurisdictions have contributed, in consultations from October 2004 to January 2005.

Project Reference Group and Project Officer

The Public Health Classifications Project Reference Group is chaired by:

Louisa Jorm, NSW Dept of Health and NPHPG representative on the National Health Performance Committee.

Reference Group members:

Michael Ackland (Department of Human Services, Victoria),

Andrea Casasola / Jackie Steele (Queensland Health),

Jenny Cleary, Pam Gollow (Department of Health and Community Services, NT),

Charles Guest (ACT Health),

Paul Jelfs (SA Department of Health),

Paul Magnus and Catherine Sykes, Robert Van der Hoek (Australian Institute of Health and Welfare (AIHW)),

Dean Martin / Sally Goodspeed (Australian Bureau of Statistics),

Karen Roger (National Public Health Partnership Secretariat),

Colin Sindall (Population Health Division, Australian Government Department of Health and Ageing),

Judy Straton, (Dept of Health WA); and

Tony Woollacott (Expenditure Project representative, SA Department of Health; Chair, National Public Health Expenditure Project Technical Advisory Group).

Tim Churches, NSW Health, is the public health content and ontology technical advisor assisting the project.

Su Gruszin, Public Health Information Development Unit (PHIDU), The University of Adelaide, is the Project Officer.

List of those consulted

Consultants who attended either a workshop (September 2004) or consultations in Sydney from October 2004, Brisbane on 6 to 8 December 2004, Melbourne on 10 and 11 December 2004, Canberra on 11 December 2004, or Perth on 18 and 19 January 2005 are (in alphabetical order):

Anna Achia, A/Manager, Cemeteries and Crematoria Program, Environmental Health, Public Health Group, Rural and Regional Health and Aged Care Services, Dept of Human Services, Victoria, (DHS, Vic)

Michele Agustin, Research Grants and Conference Support Scheme, Victorian Health Promotion Foundation (VicHealth)

Owen Ashby, Manager, Aboriginal Health Branch, Environmental Health, Dept of Health WA

Brett Bell, A/Manager, Finance Administration Division, Dept of Health WA

Bill Bellew, Adjunct Professor in Health Promotion, University of Queensland, and Director, Centre for Health Promotion, NSW Health

Jan Bennett, Assistant Secretary, Rural Health and Palliative Care Branch, Australian Government Department of Health and Ageing

John Biviano, Director, Research Workforce and Tobacco Control, VicHealth

Shirley Bowen, Director, Communicable Disease Control Directorate, Dept of Health WA

Jan Bowman, Manager, Environmental Health Unit, Social and Environmental Health, Public Health Group, Rural and Regional Health and Aged Care Services, DHS, Vic

Michelle Callander, Research Workforce and Tobacco Control Unit, VicHealth

Marion Carey , Senior Medical Advisor, Social and Environmental Health, Public Health Group, Rural and Regional Health and Aged Care Services, DHS, Vic

Ching Choi, Australian Institute of Health and Welfare (AIHW)

Jim Codde, Principal Epidemiologist, Health Information Centre, Dept of Health WA

Rebecca Conning, Research Workforce and Tobacco Control Unit, VicHealth.

Stephen Corbett, Director, Centre for Public Health, Western Sydney Area Health Service

Jim Dodds, Director of Environmental Health, Dept of Health WA

Sophie Dwyer, Director, Environmental Health Unit; Queensland Health

Emma Ellis, Senior Portfolio and Policy Officer, Child and Community Health Division, Dept of Health WA

Dinah Fleming, Dept of Health WA

Liz Geelhoed, Senior Policy Officer, Genomics Directorate, Dept of Health WA

Marcelle George, A/Principal Policy Officer, State-Commonwealth Relations Unit, Dept of Health WA

Billie Giles-Corti, Associate Professor, School of Population Health, University of WA

John Glover, Director, Public Health Information Development Unit, The University of Adelaide

Brian K Harrison, Director, Budget and Review Section, Population Health Division, Australian Government Department of Health and Ageing

Jane Heyworth, Environmental Epidemiologist, School of Population Health, University of WA.

David Hunter, Director, Classifications and Data Standards, Australian Bureau of Statistics

Jim Hyde, Director, Health Policy Unit, Royal Australasian College of Physicians

Tony Hynes, National Public Health Expenditure Project, AIHW

Ray James, Consultant, Health Promotion Directorate, Dept of Health WA

Monica Kelly, Manager, Partnership Development, Strategic Support, Public Health Group, Rural and Regional Health and Aged Care Services, DHS, Vic

Rosemary Lester, Manager, Communicable Diseases, Public Health Group, Rural and Regional Health and Aged Care Services

Kathleen Lilley, Centre Manager, Queensland Centre for Public Health

Vivian Lin, Professor of Public Health and Head of School, School of Public Health, La Trobe University, Victoria

Stephen Lodge, Manager, Legislation Review Section, Public Health Group, Rural and Regional Health and Aged Care Services

Alan Lopez, Professor of Medical Statistics and Population Health Head, School of Population Health, University of Queensland

Richard Lugg, Medical Advisor's Office, Dept of Health WA

Ian Matthews, Environmental Health, Queensland Health

Jennifer MacDonald, Manager, Food Safety and Water Regulation, Social and Environmental Health, Public Health Group, Rural and Regional Health and Aged Care Services, DHS, Vic

Richard Madden, Director, AIHW

Barbara Mouy, Director, Health Promotion Innovations Unit, VicHealth

Jan Norton, Director, Social and Environmental Health, Public Health Group, Rural and Regional Health and Aged Care Services, DHS, Vic

Alan Philp, Australian Government Dept of Health and Ageing

Isabel Redfern, Clinical Nurse Manager, North Metro Health Service, Dept of Health WA

Therese Robinson, Manager, Public Health Development, Housing Primary and Complex Care, Eastern Metropolitan, DHS, Vic

Elizabeth Rohwedder, Assistant Director, Resource Management Division, Dept of Health WA

Pauline Sanders, Manager, Executive Support, Strategic Support, Public Health Group, Rural and Regional Health and Aged Care Services

Christine Selvey, Manager, Prevention and Perinatal Health, Public Health Group, Rural and Regional Health and Aged Care Services, DHS, Vic

Mary Sheehan, Director, Centre for Accident Research and Road Safety - Queensland, Queensland University of Technology

Vic Siskind, Centre for Accident Research and Road Safety - Queensland, Queensland University of Technology

Terry Slevin, Director of Education and Research, Cancer Council WA

Merran Smith (Director, Health Information Centre, Dept of Health WA and Co-Chair, National Public Health Information Working Group

Jackie Steele, A/Executive Director, Public Health Services Branch, Queensland Health

Don Stewart, Director, Queensland Centre for Public Health

Francois Tsafack, Senior Project Officer Evaluation, Health Promotion Innovations Unit, VicHealth

Cathy Turner, Director of Research, Nursing Program, University of Queensland

Irene Verins, Project Officer, Mental Health and Wellbeing Unit, VicHealth

Andrew Wilson, Head, Division of Health Systems Policy and Practice, School of Population Health, University of Queensland

The continuing support of Richard Madden, and the additional assistance of John Goss, Tony Hynes, Daniel Aherne and Justine Boland of AIHW is gratefully acknowledged.

Thank you!

On behalf of the project, a big thank you to all who made time to engage with the public health classification, and for your perspectives, reactions and suggestions for improvement.

An example agenda and work-in-progress documentation used in consultations are shown in the following pages.

NATIONAL PUBLIC HEALTH PARTNERSHIP

Public Health Classifications Project

Consultation on Thursday 10th December, 10-12am

Consultation Objective: to meet with content experts to model a unified public health classification that is useful and useable for multiple purposes.

Consultation hosted by: Dr Michael Ackland (Member of the Public Health Classifications Project Reference Group) and Su Gruszin (Project Officer)

Agenda

10 am Introduction, background and **purposes of consultation**:

1. to model the main axes and the top levels of a unified public health classification, and
2. to identify practical uses for the classification.

10:15am **1 Model the main axes**

(a) Main public health axes in scope – are there important omissions?

(b) Public health functions and the top levels of other axes of interest – are these sufficiently captured?

2 Identify practical uses

(a) Practical example: public health expenditure reporting

(b) Other practical uses of the public health classification - how best to develop the classification to support the needs of potential users?

12 noon Close: Thanks to participating experts and close of workshop.

Material provided:

1. Project Introduction (2 pages)
2. PHCP Work-in-Process (5 pages) – includes definition of public health, general and specific uses for a public health classification, main public health axes, definitions of public health functions, and selected views.

A Background Paper is available from the project website: http://www.nphp.gov.au/workprog/phi/index.htm

NATIONAL PUBLIC HEALTH PARTNERSHIP

Public Health Classifications Project: Work-in-process

Scope, domain, focus questions and top levels of the public health classification

What is the domain that the classification will cover?	Public health.<br><br>**Definition**: Public health is the organised response by society to protect and promote health, and to prevent illness, injury and disability. The starting point for identifying public health issues, problems and priorities, and for designing and implementing interventions, is the population as a whole, or population sub-groups. (NPHP 1998)<br><br>**Principles**: The classification should be inclusive, and deliberately broad at the top classes.
For what are we going to use the classification?	**Generally**, to develop a broad, generalisable public health classification that can be used to:<br>▪ organise information to facilitate answering key public health questions e.g. expenditure on prevention of obesity;<br>▪ reflect the full scope and breadth of public health activity, in expenditure and performance indicator reporting;<br>▪ articulate, describe and define public health, and promote consistency in describing public health (eg through standardised instructions);<br>▪ build in specific content expertise in different areas of public health;<br>▪ relate to other high level models of health (eg through interface and reference terms);<br>▪ structure and design information/communications e.g. in websites or report chapters.<br><br>**Specifically**, a public health classification could be used to:<br>▪ promote standardised definitions, terminology and reporting of public health and public health functions to improve accountability across jurisdictions, eg through the development of a national Public Health Report describing public health in Australia;<br>▪ build systems such as a web-based database of public health projects that allows routine, bottom up, multi-dimensional reporting of public health projects;<br>▪ create semantic web documents that are 'marked up' for meaning (for the Semantic Web, the next generation of the world wide web) and which can be understood and manipulated by computers (e.g. computer agents can trawl semantic web documents for information to answer questions, eg what is the project expenditure, how many people work on the project, in what settings?).

For what types of questions should the classification provide answers?	Sample focus questions include:<br>▪ How much was spent on prevention of obesity? Other 'advocacy-type' questions, e.g. difference in expenditure on prevention of HIV/AIDS relative to other preventable diseases, relative expenditure on specific risk factors or diseases?<br>▪ Has health funding to preventive or promotive investments increased?<br>▪ What is public health?<br>▪ How is public health relevant to components of the human services delivery system?<br>▪ Why do public health unit costs differ across jurisdictions?<br>▪ Can we describe screening in clinical settings eg GP surgeries for pap testing?<br>▪ What did we invest in social marketing last year?<br>▪ Can we replicate the output of other models? (eg Public Health Expenditure Reporting, public health component of OECD Health Accounts)

Top-level public health classes

The top-level public health classes listed for examination, some of which have been examined in more detail to date, are:

TOP-LEVEL PUBLIC HEALTH CLASSES	Working definition
FUNCTIONS	Public health functions. The purpose of public health actions/activities/interventions/ programs; why public health does what it does.
HEALTH & WELL-BEING ISSUES	Health and well-being issues/ concerns/ topics. Comprises: Well-being, Disease, Injury, Disability/ Functioning. Relates to definition of health as "A state of complete physical, mental and social well-being and not merely the absence of disease or infirmity." (WHO)
DETERMINANTS OF HEALTH	Population level determinants of health. Determinants of health are factors which influence health status and determine health differentials or health inequalities.
METHODS	Methods, approaches, systems, techniques, modes of procedure, operation or production; the methods or ways by which the functions of public health are carried out.
BODIES OF KNOWLEDGE	Bodies of knowledge (BoK), methods & tools used by public health (PH). Contains those tools that are specific to PH & those that are general (non PH specific) and are used by PH.
POPULATION GROUPS	Populations and subpopulations, as defined in terms of other classes.
SETTINGS	Milieux in which public health activities and interventions are carried out, institutional and social settings, partnerships, eg schools, local councils, hospitals, workplaces...
RESOURCES	Resources. 'The means available for the operation of health systems, including human resources, facilities, equipment and supplies, financial funds and knowledge'. Includes person-time and calendar time.

A further high level class, CONTEXTUAL FACTORS has been suggested. This is currently defined as 'Contextual or macro- environmental factors that could be included in environmental scanning, for example, 'the Economy, Government, Legal, Technology, Ecology, Socio-cultural (including demographic factors, attitudes and cultural structures), and Potential Suppliers.'[72]

Public health functions

Subclasses of the top-level public health class FUNCTIONS are scoped as follows:

**FUNCTIONS**	Public health functions. The purpose of public health actions/interventions/interventions/ programs; why public health does what it does.
Assess health of populations	Assess health of populations. 'Assess, analyse and communicate population health needs and community expectations'.[73] Includes macro-environmental scanning.
Promote better health	Promote better health, promote well-being. 'Promote better health through promoting, supporting and encouraging healthy lifestyles & behaviours, through action with individuals, families, communities and wider society'.[74]
Prevent disease/disability/injury<br>Incl: Prevent initial occurrence disease/ disability/ injury, & Early detection of disease/ disability/ injury	Prevent initial occurrence of disease, disability or injury. Measures to prevent occurrence of disease include e.g. risk factor reduction, and Early detection of disease, disability, and injury.
Maintain health & QoL<br>Incl: Prevent/ reduce progression/ recurrence, & Mitigate effects of disease/ disability/ injury]	Maintain health and quality of life by preventing the progression or mitigating the effects of established/existing disease, disability, injury. Health maintenance addresses quality of life, secondary/tertiary prevention (eg palliative care), and/or public health as a social good (e.g. methadone programs that mitigate effects on society rather than treating dependence/ addiction). Includes working with vulnerable populations eg drug addicts, people with terminal illness.
Protect from & prevent threats to health	Protect from, and prevent, external threats to health. Respond to major public health threats, including bio-terrorism and naturally occurring epidemics. Includes: Manage public health aspects of disasters; Respond to and mitigate threats to health. [Health protection]
Maintain/develop PH Infrastructure	Maintain and develop investment in and maintenance of PH infrastructure, eg sewerage plants, road surfaces, laboratories, PH informatics & other systems.

[72] Wikimedia Foundation 2004.

[73] NPHP 2000: 2.

[74] NPHP 2000: 2.

Selected views of the classification including a practical example from public health expenditure reporting, and another from the UK, follow.

View 1

showing the main public health classes captured in the classification

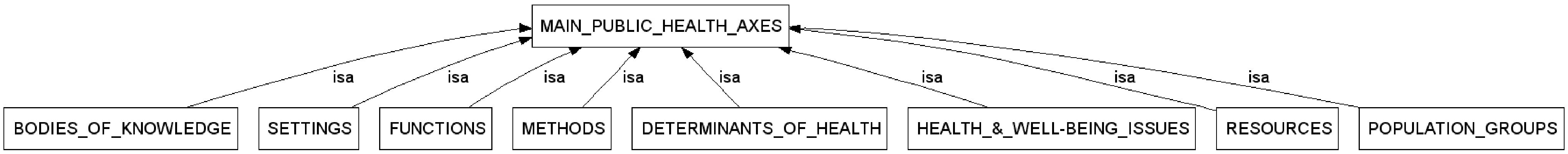

View 2 A practical example showing classes (above) used to classify 'public health activities' (PH_activities)

(public health activities derived from Public Health Expenditure Reporting and input from the Reference Group)

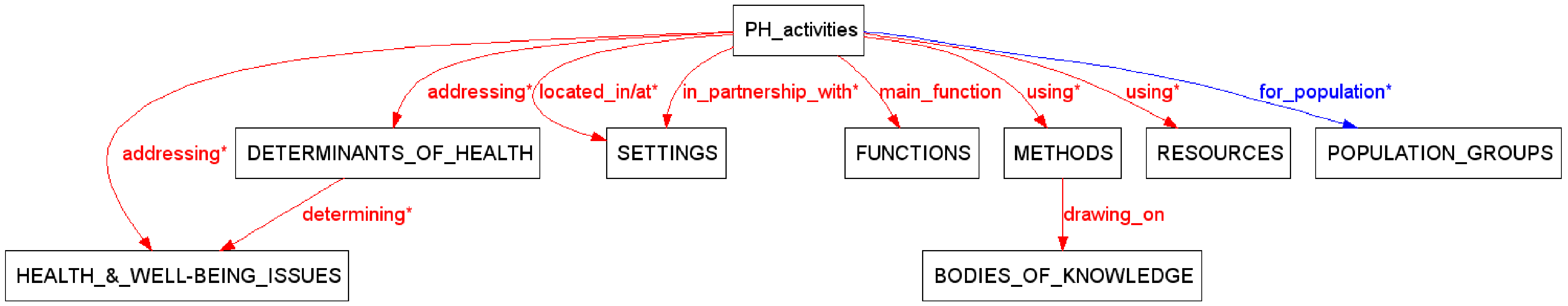

View 3 showing example data 'public health activities' described by various classes

('io' stands for 'instance of')

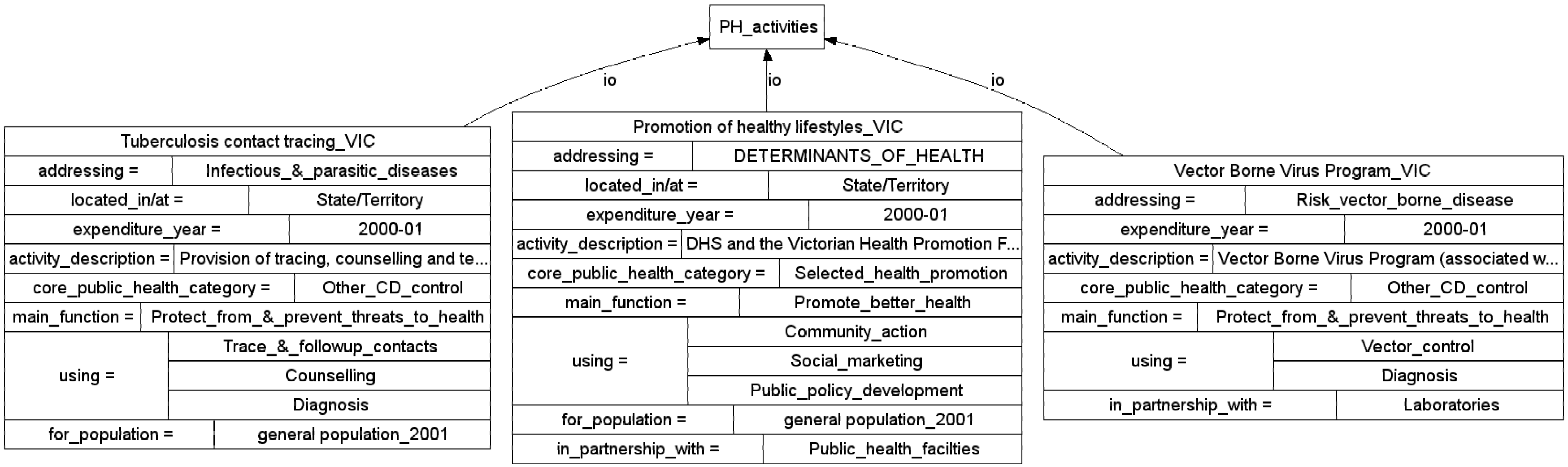

Developments in public health classification in the UK

Related recent developments in the UK include the development of a Public Health Information Tagging Standard –to provide website access to public health resources – and a National Public Health Language, incorporating other thesauri and vocabularies to improve web-based searching and retrieval for public health resources.

A Public Health Information Tagging Standard

A web-based system for the classification and retrieval of public health resources was conceived by Julian Flowers of the Eastern Region Public Health Observatory (ERPHO) in the UK, as there was no system specifically suitable for this purpose. The Public Health Information Tagging Standard (PHITS) was borrowed categories from a number of extant sources,[75] and took contributions from public health specialists nationwide(see *Figure 10)*.

Figure 10 Using the UK Public Health Information Tagging Standard

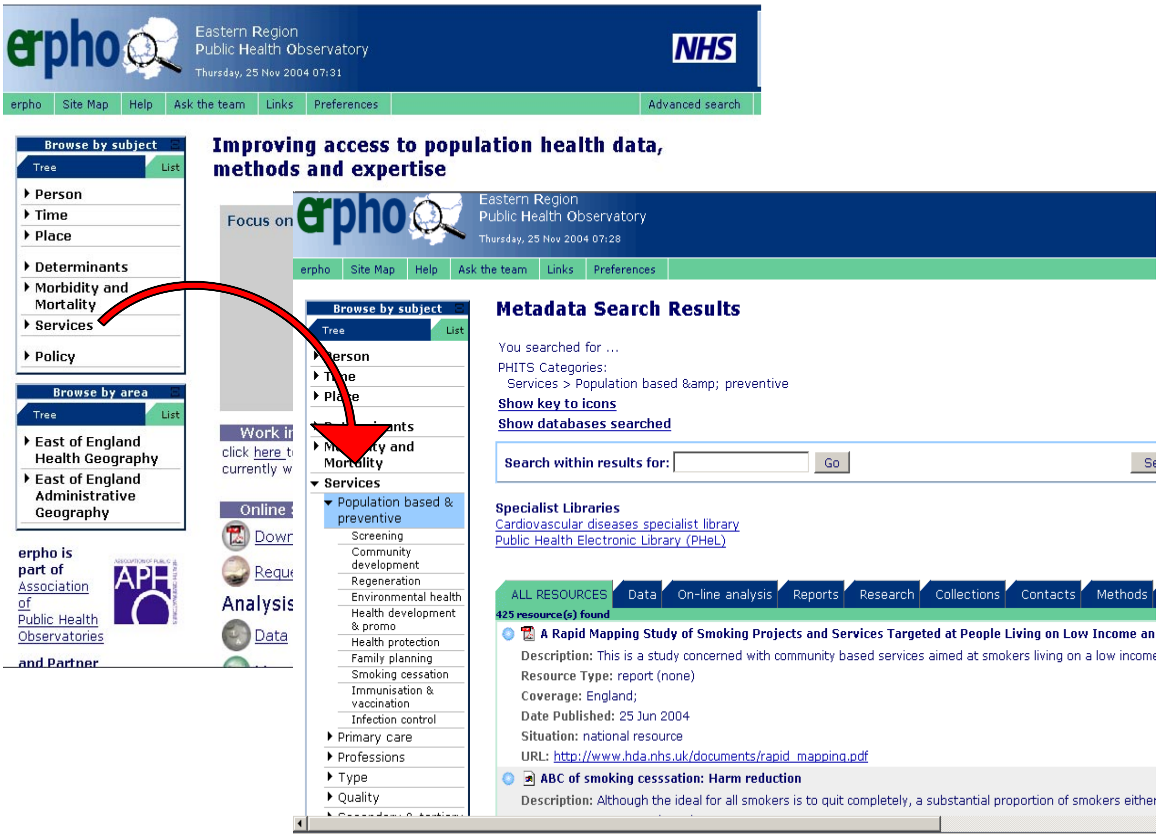

Source: Eastern Region Public Health Observatory www.erpho.org.uk accessed November 2004.

Figure 10 shows PHITS describing public health resources on the website of the ERPHO. Subjects or classes of interest can be selected from the 'Browse by subject' box on the left side of the underlying screen print. The overlying screen print shows the 'Services' class and its finer subclasses (e.g. 'Population based and preventive', 'Primary care'). The tabbed entries to the right show the types of resources available (e.g. all resources, data), and provides typical information on individual resources (e.g. 'A rapid mapping study of smoking

[75] Sources included ICD10, MeSH, and SNOMED.

projects', an 'ABC of smoking cessation'), including the URL of the resource for instant access.

After its introduction on the ERPHO website, PHITS was adopted as a standard for use by all ten Public Health Observatories in England and Wales, as well as other public health organisations, such as Public Health Ireland.[76] Initially intended purely as a web site categorisation and retrieval system, PHITS has now become part of the development of a National Public Health Language for the UK.

A National Public Health Language for the UK

PHITS has been integrated with the UK Health Development Authority's Public Health Information Thesaurus[77] and two other controlled vocabularies, to create the National Public Health Language (NPHL) for the UK (*Figure 11)*. The development of a common public health language is intended to facilitate interoperability and improve the efficiency of searching for and retrieving, public health information and resources held on websites and in databases. All organisations that were already using PHITS have agreed to move to the NPHL when version one was available (December 2004[78]). NPHL users will have both a public health biased classification system; and a powerful, thesaurus-driven, categorisation and searching mechanism for use on web sites.[79] *Figure 11* shows the entry website for online access to the NPHL (left side) and top-level classes and their definitions (right side).

Figure 11 UK National Public Health Language including top-level classes

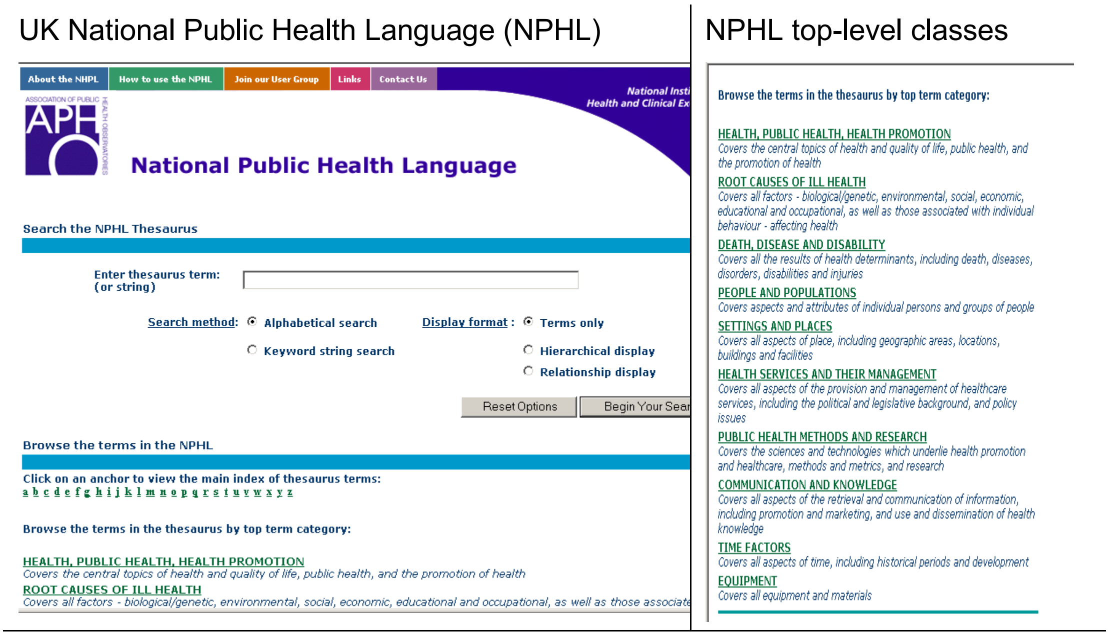

Source: National Institute for Health and Clinical Excellence (UK) http://www.nphl.nhs.uk/index.html accessed August 2005. The NPHL is also available as hierarchical and alphabetical lists.

---

[76] Information from Julian Flowers and Peter Cornelissen, Eastern Region Public Health Observatory, Cambridge, UK. The website of the ERPHO is at www.erpho.org.uk.

[77] The HDA's Public Health Information Thesaurus (based on the European Multilingual Thesaurus on Health Promotion) was used to index book and journal resources on evidence-based public health, health promotion, and health inequalities in HealthPromis (the health promotion bibliographic catalogue), the HDA's Evidence Base, and the Public Health Electronic Library.

[78] More information on *Interoperability in Public Health and the NPHL* see http://www.nphl.nhs.uk/conference.html.

[79] These developments coincide with that of the electronic Government Metadata Standard (e-GMS), derived from the Dublin Core, with the NPHL used as the subject category metadata element (Peter Cornelissen, ERPHO).

Appendix C: Defining public health

The Reference Group debated and adopted the National Public Health Partnership definition of public health as an appropriate definition for the domain to be covered in the development of a public health classification.

**Domain to be covered by the classification:**	Public health.<br><br>**Definition**: Public health is the organised response by society to protect and promote health, and to prevent illness, injury and disability. The starting point for identifying public health issues, problems and priorities, and for designing and implementing interventions, is the population as a whole, or population sub-groups.<br><br>Source: National Public Health Partnership (1998) Public Health in Australia: The Public Health Landscape: person, society, environment. Melbourne: NPHP.

An alternative definition 'Public health activities aim to benefit populations and tend to emphasise prevention, protection and health promotion as distinct from treatment and other interventions that are tailored to individuals' (AIHW 2004, revised) was discussed but not agreed. The inclusion of a specific reference to measuring or achieving outcomes was considered implicit in the Partnership definition (above).

Key points in the discussion (detailed below) included whether treatment of individuals should be included, differences in individual health versus public health benefit, and whether the definition should be inclusive or exclusive.

Potential boundaries around treatment and diagnosis were raised. It was suggested but not agreed, that only treatment of asymptomatic (including undiagnosed) individuals should be included in the definition of public health, with the underpinning concept of *delivery* at population (rather than individual) level. The rebuttal example of public health activities in drug and alcohol treatments of individuals which also led to population or social good (eg 'harm minimisation') was acknowledged.

'*[T]he starting point for identifying public health issues …*' was discussed as meaning the initial conceptualisation of public health (e.g. design of SNAP [smoking, nutrition, alcohol and physical activity] population treatment guidelines for GPs[80]). There is no clear boundary at the top level between public health benefit and individual health benefit, and there are crossover benefits between individuals and populations in many public health areas, e.g. immunisation for communicable diseases protects the individual and contributes to population ('herd') immunity; SNAP guidelines prepared from a population health perspective are used by GPs to treat individuals. It was agreed that public health delivery, and outcome, can be at individual as well as population levels.

---

[80] The Royal Australian College of General Practitioners *SNAP Guide* (RACGP 2004) identifies harm associated with smoking, poor nutrition, excess alcohol consumption and a sedentary lifestyle – behavioural risk factors that affect the health of the Australian community. The *Guide* assists GPs systematically target patients and offer treatment options appropriate to their needs. See http://www.racgp.org.au/guidelines/snap/.

The suggestion to include in the Partnership definition, the words '*and measuring outcomes*', or '*to achieve outcomes in*' was discussed but not agreed, as outcomes were considered to be implicit in the definition, and did not need to be spelled out.[81]

The difference in the definition of public health used by the AIHW to that used by the Partnership was noted, and thought to reflect an attempt to differentiate between treatment (of individuals) versus prevention activities directed towards populations, e.g. organised screening programs. There was however, a preference for an inclusive - rather than exclusive, or rule-based - definition, as the nature of public health was considered to be inclusive by definition. It was agreed that the type of distinctions discussed above (individual versus population, treatment versus non-treatment) could be made at other levels of the class hierarchy, and relative to the purpose of using the classification (i.e. in specific applications), rather than at the top level, which should be broad and inclusive rather than defined by exclusions.

Although concerns about wording remained, any likely changes were deemed minor, and the Partnership's original definition (as shown in the box) was agreed.

[81] With these inclusions the definition would read: 'Public health is the organised response by society to protect and promote health, and to prevent illness, injury and disability. The starting point for identifying public health issues, problems and priorities and for designing, implementing and evaluating interventions, and measuring outcomes, is the population as a whole, or population sub-groups' (NPHP 1998, adapted).

Appendix D: Contextual drivers and technical context

Contextual drivers for the project include the conceptualisation of public health as a complex and 'wicked problem', and the desirability of positioning the classification of public health to take advantage of near future developments in the Semantic Web and in computable information created by ontology-development software (these terms are defined in the *Glossary*).

Context:

- Conceptualising public health – no 'right' or 'wrong' – best that can be achieved is consensus of experts that it is good enough.
- Near future developments - Semantic Web, computable information created using ontologies and standards.

The question of why the project was using an ontology to develop a classification came up repeatedly and is addressed below. An ontology is an explicit formal specification of the concepts in a domain (in this case, public health), their attributes and the relations among them, which allows people to share a common understanding of the structure of information.[82] It defines a common vocabulary for people who need to share information, and can be used to produce machine-readable definitions of the basic concepts in the domain and relations among them. Ontologies are becoming widely used in Web-based applications, to make existing information more available and better connected.

A major reason for developing a public health classification as an ontology is to exploit the near future capabilities of this connectedness on the Semantic Web. The Semantic Web is the enhancement occurring now, of the World Wide Web into 'a global database', with infrastructure that allows machines as well as humans to process meaningful information.[83] Ontologies have the facility to produce machine-readable data that includes both the structure and the information classified by it, while the Web infrastructure means information need no longer be held centrally to be centrally accessible.

Ontologies can be developed in a top down, or bottom up manner, or using a combination of both. There is 'no correct way to model a domain – there are always viable alternatives' and ontology development is 'necessarily an iterative process'. Practical applications and discussions with content experts are used to 'evaluate and debug', leading to revisions of the

---

[82] Noy & McGuinness 2001. For more information on ontologies and the Semantic Web developments, see the *Glossary*.

[83] The Semantic Web and computable information are the visions of Tim Berners-Lee, the creator of the World Wide Web (familiar to us through search engines like Google). The future Web is viewed as a web of data, 'like a global database', where information has 'well-defined meaning, better enabling computers and people to work in cooperation'. Making information on the Web 'semantic' (meaningful) means more efficient searching as if it is 'one giant database, rather than one giant book' (Berners-Lee 1998). Semantic Web infrastructure will allow machines as well as humans to make deductions and organise information. The approach is to develop languages that express information in machine processable forms. Architectural components include semantics (meaning of elements), structure (organisation of elements), and syntax (communication). Abstract representation of data is based on existing standards (eg RDF – Resource Description Framework) and standards yet to be defined, in development by the World Wide Web Consortium (W3C) collaborating with researchers and industrial partners. (Berners-Lee et al. 2001, Berners-Lee & Miller 2002)

initial ontology, in an iterative design process that continues through the whole of the ontology's lifecycle.[84] This iterative development style is a good fit for complex or wicked problems. Because public health is complex it is technically conceptualised as a 'wicked problem'[85], meaning that there is no *definitive* formulation or solution, no 'right' or 'wrong', no absolute truth or perfect solution that holds for all cases—the best that can be achieved is a consensus of public health experts that it is good enough.

In consultations it was clear that the conceptualisation of public health is time-specific (e.g. the 'old' and the 'new' public health), includes many contested definitions and terms, as well as fuzzy borders and boundaries. There is not even agreement on what it should be called, with the terms 'population health' and 'preventive health' currently challenging 'public health'.

Two principles of development (see *Section 3.1.5*) address these difficulties: be inclusive; and, set rules and boundaries in applications, rather than in the development of the classification itself.

Inclusiveness is a response to the divergence of views and definitions encountered in field consultations. The project took the position that a public health classification should not exclude elements that some (but not all) consider to be an important part of public health. It should actively seek to include divergent views since its usefulness as a unified classification depends on the best coverage of the *breadth* of public health.

Rules and boundaries can and should be determined in practical applications rather than in the ontology. For instance, for the purposes of reporting health and public health expenditure, it may be determined that all one-to-one treatment services in clinical settings are *not* public health services. Another use might determine that *some* one-to-one clinical treatments, such as those for immunisations, sexually transmitted infections, or drug detoxification, *are* public health services. The decision to set a constraint or boundary for a particular application should not preclude the wider scope of a 'public health classification', which is developed as an ontology.

A single ontology can be used to develop one or more classification systems, by developing specific rules and boundaries (developed as 'constraints' in the ontology) to organise classes into a hierarchy, and to assign elements to unique classes.

Although defining and specifying classes (concepts within the domain of interest) is central to developing an ontology, the emphasis is on modeling the relationships among classes, rather than on hierarchy (broader classes contain the more specific) or mutual exclusion (an element cannot be in more than one class).

An ontology allows elements to be assigned to more than one class. This is useful, for instance, for areas (of which there are many in public health) on which there is little agreement and competing views. In a classification system, with its emphasis on mutually discrete classes, it is not so easy to do this.

---

[84] Noy & McGuinness 2001: 4.

[85] The wicked problem concept in design was described by Rittel & Webber (1984) in the context of social planning. They pointed out that in solving a wicked problem, the solution of one aspect may reveal another, more complex problem. Ten rules define the form, including that there is no *definitive* formulation of a wicked problem (no stopping). Solutions to wicked problems are not therefore true-or-false, but good-or-bad.

Figure 12 Multiple inheritance of classes in an ontology

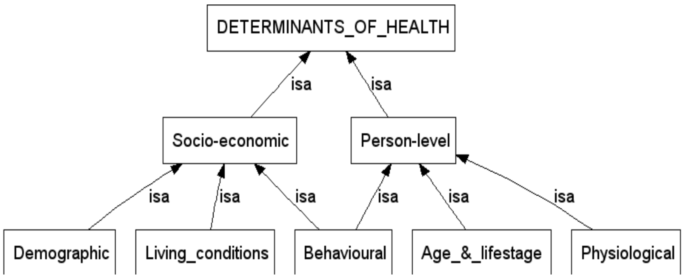

The 'isa' relation arrows show the parent class or classes that each child class belongs to in the class hierarchy of the ontology.

A concrete example is the categorisation of behavioural factors. Most public health experts would agree that as a determinant of health these contribute to health risk and/or protection; however some see behavioural factors as exclusively personal, while others see them as exclusively socio-economic, and some see them as both. Using an ontology, they can be classed under both categories, so that those who expect to find them under personal factors will do so, as will those who expect to find them under socioeconomic factors, as illustrated in *Figure 12*. Thus all are satisfied (have found the category where they expected to), a practical result has been achieved, and an indecisive argument about where it is 'rightly' to be found has been avoided.

Sophisticated software tools are available to assist in developing ontologies. These allow multiple inheritance (as described above), definition of relationships among classes, specifications of attributes of classes, and classification of elements (instances). Aspects of public health (characteristics, attributes, etc) can be described either textually as descriptions, mathematically as values, or in terms of other classes in the class hierarchy, and can be constrained by specific rules.

Ontology development software is the backbone of the next generation of information tools. Increasingly, existing classification systems are being migrated to, or developed in, ontology building software such as *Protégé*[86] (used by this project). This software makes it easy to render form and content for the Web.

As the Semantic Web develops and ontologies become more widely used in Web-based applications, the development of the public health classification in an ontology can be expected to produce major productivity gains in making existing information more available and better connected.

[86] More information on *Protégé,* and the free, open source *Protégé* software, are available from Stanford University at http://protege.stanford.edu/index.html.

Appendix E: Names: population health and public health

In some jurisdictions the term 'Population health' appears to have superceded that of 'Public health' in health departments and universities .

Table 7 Sample of departmental names in jurisdictions

Jurisdiction	Australia	AIHW	NSW	Vic	Qld	WA	SA	Tas
Departmental name used 2005	Population Health Division	Population Health	Population Health Division	Public Health	Public Health Services Branch	Population Health Division	Population Health Directorate	Population Health
Position within Dept	One of 5 Health and Ageing Sector Divisions (as opposed to Cross Portfolio Divisions)within the Dept of Health and Ageing		One of four divisions in NSW Health.	Within the Rural & Regional Health & Aged Care Services Division – one of two in the Dept of Human Services.	One of four branches in the Health Services Directorate - one of five directorates in Qld Health.	A division in the Dept of Health	A division in the Dept of Health (Dept created 1 July 2004).	Within the Community, Population & Rural Health Division, one of 6 (content) divi-sions in the Dept of Health & Human Services.
Previous name	?	Public Health	Public Health	?	?	Public Health	Public Health	?Public & Envir-onmental Health

Table 8 Sample of school names in universities

Jurisdiction	NSW	Vic	Qld	WA	SA
University 1	School of **Public Health**, Faculty of Medicine. University of Sydney	School of **Population Health,** University of Melbourne	School of **Population Health,** University of Queensland	School of **Population Health**, Faculty of Medicine and Dentistry, University of WA	Dept of **Public Health**, Faculty of Health Sciences, The University of Adelaide.
University 2	School of **Public Health & Community Medicine**, Faculty of Medicine, Uni of NSW	School of **Public Health**, Faculty of Life Sciences, La Trobe University	School of **Public Health,** Griffith University	School of **Public Health,** Curtin University.	Dept of **Public Health**, Medical School, Faculty of Health Sciences, Flinders University

+ School of **Population Health**, University of Auckland.
